# Bronchoalveolar lavage fluid cellular fractions reflect individual histopathological features in interstitial pneumonia

**DOI:** 10.1371/journal.pone.0361768

**Published:** 2026-10-30

**Authors:** Shion Miyoshi, Susumu Sakamoto, Sakae Homma, Yasuhiko Nakamura, Yusuke Usui, Hiroshige Shimizu, Muneyuki Sekiya, Naohisa Urabe, Takuma Isshiki, Yoko Azuma, Naobumi Tochigi, Kazuma Kishi

**Affiliations:** 1 Department of Respiratory Medicine, Toho University Omori Medical Center, Ota-Ku, Tokyo, Japan; 2 Department of Respiratory Medicine, Ikegami General Hospital, Otaku, Tokyo, Japan; 3 Division of Chest Surgery, Department of Surgery, Toho University School of Medicine, Ota-Ku, Tokyo, Japan; 4 Department of Surgical Pathology, Toho University School of Medicine, Ota-Ku, Tokyo, Japan; Novartis Institutes for BioMedical Research, UNITED STATES OF AMERICA

## Abstract

Bronchoalveolar lavage fluid (BALF) cellular analysis is an adjunctive diagnostic tool in interstitial pneumonia (IP), yet its relationship with individual histopathological features remains unclear. We retrospectively compared BALF cellular fractions with 24 histopathological features in 67 IP patients who underwent BALF and surgical lung biopsy at a single center (2005–2020). False discovery rate correction was applied (*q* < 0.05), followed by multivariable logistic regression and Kaplan–Meier analysis. BALF lymphocyte proportion was negatively associated with fibroblastic foci (13.0% vs 29.0%, *q* = 0.002) and positively with bronchiolitis (*q* = 0.029), intraluminal organization (*q* = 0.002), and epithelioid cell granulomas (*q* = 0.012). On multivariable analysis, fibroblastic foci were independently associated with a lower likelihood of BALF lymphocytosis (odds ratio 0.23, p = 0.018), and this association persisted after exclusion of patients with a usual interstitial pneumonia (UIP) or probable UIP pattern and of patients with idiopathic pulmonary fibrosis. Intraluminal lymphocyte proliferation was also associated with BALF lymphocytosis, although this estimate was based on few patients. Patients with fibroblastic foci had poorer survival (*p* = 0.003), whereas survival did not differ when patients were stratified by BALF lymphocyte proportion. ROC analysis identified a BALF lymphocyte cutoff of 18.5% for the presence of fibroblastic foci (AUC 0.76), and adding BALF lymphocyte proportion to the high-resolution computed tomography pattern improved discrimination of fibroblastic foci (AUC 0.847 versus 0.686). Neutrophil and eosinophil proportions showed no significant associations after correction. BALF lymphocyte proportion is associated with several histopathological features in IP, most strongly with fibroblastic foci, and may provide complementary information where surgical lung biopsy is not feasible.

## Introduction

Idiopathic interstitial pneumonias comprise a heterogeneous group of respiratory diseases encompassing multiple subtypes each characterized by distinct pathological involvement of the pulmonary interstitium. Idiopathic pulmonary fibrosis (IPF), in particular, is associated with a poor prognosis and represents a progressive chronic fibrosing interstitial lung disease (ILD), characterized histopathologically by a usual interstitial pneumonia (UIP) pattern [1,2]. Other ILD subtypes include nonspecific interstitial pneumonia (NSIP) and connective tissue disease-associated ILD (CTD-ILD). In addition, unclassifiable interstitial pneumonia has been described, reflecting overlapping features of UIP and NSIP patterns [1,3]. In such atypical or unclassifiable interstitial pneumonia (IP), definitive disease classification by subtype requires careful evaluation through comprehensive multidisciplinary discussion (MDD) [1].

Surgical lung biopsy (SLB) is considered the gold standard for pathological diagnosis in patients with ILD, however its high invasiveness remains a major concern. More recently, transbronchial lung cryobiopsy has been employed as an alternative when SLB is not feasible, although it carries a risk of complications such as bleeding, and its diagnostic utility remains controversial [4,5]. For patients in whom invasive diagnostic procedures cannot be performed, findings from analysis of bronchoalveolar lavage fluid (BALF) obtained via bronchoscopy are often used as complementary diagnostic information alongside radiological and clinical features.

BALF cellular analysis is recommended as an adjunctive diagnostic tool in ILD, and its clinical utility has been reported in several disease entities, including hypersensitivity pneumonitis and sarcoidosis [6–10]. In addition, prior studies have suggested that BALF cellular fractions may have prognostic value in patients with IPF [11]. and that an increased BALF lymphocyte proportion may reflect steroid responsiveness in ILDs other than IPF [12]. Collectively, these findings support the role of BALF cellular fractions as a valuable adjunct in the diagnostic evaluation and clinical management of ILD.

Despite the recognized utility of BALF in ILD, the relationship between BALF cellular profiles and individual lung histopathological features has not been well defined. Previous studies examining BALF in ILD have largely focused on comparisons across pathological patterns or on establishing final diagnoses [6–8,10,13,14], rather than detailed systematic evaluation of associations between specific lung histopathological features and BALF cellular fractions. In this study, we comprehensively assessed lung histopathological features obtained by SLB in patients with IP and comparatively examined their associations with BALF cellular fractions obtained via bronchoscopy in the same individuals. The aim of this study was to clarify the relationship between pulmonary histopathological features and BALF findings in patients diagnosed with IP. If BALF cellular profiles are associated with individual lung histopathological characteristics, BALF analysis could provide complementary information about those features, particularly in unclassifiable IP, where guideline-based pattern classification is not attainable and definitive diagnosis therefore remains challenging.

## Methods

### Patients

This single-center retrospective study was conducted to evaluate associations between distinctive lung histopathological features and BALF cellular fractions in patients with IP. The study protocol was approved by the Ethics Committee of Toho University Omori Medical Center (Approval No. M23236) in accordance with Japanese regulations. The requirement for informed consent was waived per the retrospective nature of the study. Information regarding the study was disclosed on the institutional website, and patients were provided the opportunity to decline participation via an opt-out process. The study was conducted in accordance with the principles of the amended Declaration of Helsinki. Data were collected from the medical records for research purposes during April 2024. During data collection, the authors had access to information that could identify individual participants, such as patient identification numbers; thereafter, the dataset was fully anonymized prior to analysis.

Overall, 98 patients who underwent both BALF analysis and SLB for evaluation of ILD at Toho University Omori Medical Center, Japan between July 2005 and January 2020 were initially identified. We excluded 7 patients with missing BALF data, 1 with missing SLB data, 1 due to BALF analysis failure, 6 with disease progression between BALF and SLB, and 16 whose final diagnosis was not IP. Consequently, 67 consecutive patients were included in the final analysis (Table 1).

**Table 1:** Patient characteristics.

Variable	Value
Patients, *n*	67
Sex, male/female, *n*	34/33
Age, years (median, range),	64 (38–85)
Smoking history, Never/Former/Current, *n*	32/23/12
Treatment at the time of BALF or SLB, *n*	None
Interval between SLB and BALF, days (median, IQR)	43 (18–75)
Laboratory data (median, IQR)	
LDH, U/L	251 (222–282)
KL-6, U/mL	1061 (625–1511)
SP-D, ng/mL	160 (87.4–277)
Pulmonary function test results (median, IQR)	
FVC, mL	2430 (1990–3130)
%FVC	90 (75.5–104.2)
DLCO, % predicted	73.1 (55.9–85.5)
BALF analysis (median, IQR)	
BALF recovery rate, %	53 (47–61)
Total cell count, × 10^5^/mL	1.0 (0.7–1.7)
Macrophages, %	68 (34–79)
Neutrophils, %	4.0 (1.0–7.0)
Lymphocytes, %	21 (8.0–34)
Eosinophils, %	3.0 (1.0–6.0)
Final diagnosis, n	
IPF	9
Idiopathic NSIP	19
Unclassifiable IP	23
CTD-ILD	9
CHP	7
HRCT pattern, n	
Probable UIP	10
Indeterminate for UIP	12
Alternative diagnosis	45
Pathological pattern, n	
UIP	2
Probable UIP	9
Indeterminate for UIP	12
Alternative diagnosis	44

The interval between SLB and BALF is reported as an absolute value, since SLB preceded bronchoalveolar lavage in 2 patients. BALF, bronchoalveolar lavage fluid; SLB, surgical lung biopsy; IQR, interquartile range; LDH, lactate dehydrogenase; FVC, forced vital capacity; DLCO, diffusing capacity of the lung for carbon monoxide; IPF, idiopathic pulmonary fibrosis; NSIP, nonspecific interstitial pneumonia; IP, interstitial pneumonia; CTD-ILD, connective tissue disease–associated interstitial lung disease; CHP, chronic hypersensitivity pneumonitis; HRCT, high-resolution computed tomography; UIP, usual interstitial pneumonia.

### Bronchoscopic procedure and BALF analysis

A standard flexible fiber-optic bronchoscope (BF-1T260; outer diameter, 5.9 mm; Olympus, Tokyo, Japan) was used in all patients. Following inspection of the airway, the bronchoscope was wedged into a specific segmental bronchus within the affected lung region, or alternatively into the lingular segment or right middle lobe. Sterile saline was then instilled in 2–4 aliquots of 50 mL each, yielding a total instilled volume of 100–200 mL. After each instillation, BALF was gently aspirated to minimize airway collapse. BALF samples were processed by density-gradient centrifugation using mononuclear cell preparation tubes with sodium heparin (BD vacutainer; Becton Dickinson and Company, NJ). Cytocentrifuged smears were stained with Wright-Giemsa stain for differential cell counts. T-cell phenotyping of BALF lymphocytes was performed using flow cytometric analysis.

### Radiological and pathological assessment

Routine computed tomography (CT) scanning of the entire lung was performed using a slice thickness of 5–10 mm. All patients additionally underwent thin-section high-resolution CT (HRCT) with a slice thickness of 1.0 mm for detailed assessment of lung parenchymal abnormalities. Obtained images were reconstructed using standardized window settings and independently evaluated by three pulmonologists (S.M., S.S., and S.H.), who were blinded to the patients’ clinical information. HRCT patterns were classified into four categories: UIP, probable UIP, indeterminate for UIP, and alternative diagnosis, according to the American Thoracic Society/ European Respiratory Society/ Japanese Respiratory Society/ Latin American Thoracic Association (ATS/ERS/JRS/ALAT) statement [1].

Lung tissue specimens were obtained by SLB from at least two different sites. Histopathological evaluation was performed on hematoxylin–eosin–stained and Elastica van Gieson–stained sections. Histopathological patterns were classified using the same ATS/ERS/JRS/ALAT categories: UIP, probable UIP, indeterminate for UIP, and alternative diagnosis [1]. In addition, 24 lung histopathological features were systematically assessed: alveolitis, fibrosis, fibroblastic foci, subpleural and perilobular distribution, bronchiolitis, intraluminal organization, bronchiectasis, honeycombing, epithelioid cell granulomas, bronchovascular bundle thickening, lymphoid hyperplasia, lymphoid follicles, vascular endothelial hypertrophy, intraluminal macrophage proliferation, intraluminal neutrophil proliferation, intraluminal eosinophil proliferation, intraluminal lymphocyte proliferation, intraluminal plasma cell proliferation, type II pneumocyte hyperplasia, bronchial epithelial cell hyperplasia, pleural disease, airspace enlargement with fibrosis, emphysema, and bullae. Each of the 24 features was recorded as present or absent, since binary assessment was expected to yield greater consistency between evaluators than graded scoring. All 24 features were independently reviewed by a pathologist (S.H.) and a pulmonologist with pathology training (Y.N.), both blinded to clinical information, and the final pathological diagnosis was determined by consensus between these two evaluators.

The presence or absence of each of the 24 histopathological features (Table 2) was examined and compared with BALF cellular fractions. In addition, BALF cellular fractions were analyzed comparatively to both pathological tissue patterns and HRCT imaging patterns.

**Table 2:** Pathological features in patients with interstitial pneumonia (*n* = 67).

Pathological feature(s)	Present *n* (%)	Absent *n* (%)
Alveolitis	62 (92.5)	5 (7.5)
Fibrosis	62 (92.5)	5 (7.5)
Fibroblastic foci	40 (59.7)	27 (40.3)
Subpleural and perilobular distribution	37 (55.2)	30 (44.8)
Bronchiolitis	17 (25.4)	50 (74.6)
Intraluminal organization	19 (28.4)	48 (71.6)
Bronchiectasis	56 (83.6)	11 (16.4)
Honeycombing	6 (9.0)	61 (91.0)
Epithelioid cell granulomas	6 (9.0)	61 (91.0)
Bronchovascular bundle thickening	52 (77.6)	15 (22.4)
Lymphoid hyperplasia	56 (83.6)	11 (16.4)
Lymphoid follicle	28 (41.8)	39 (58.2)
Vascular endothelial hypertrophy	3 (4.5)	64 (95.5)
Intraluminal macrophage proliferation	7 (10.4)	60 (89.6)
Intraluminal neutrophil proliferation	10 (14.9)	57 (85.1)
Intraluminal eosinophil proliferation	6 (9.0)	61 (91.0)
Intraluminal lymphocyte proliferation	16 (23.9)	51 (76.1)
Intraluminal plasma cell proliferation	7 (10.4)	60 (89.6)
Type II pneumocyte hyperplasia	61 (91.0)	6 (9.0)
Bronchial epithelial cell hyperplasia	59 (88.1)	8 (11.9)
Pleural disease	3 (4.5)	64 (95.5)
Airspace enlargement with fibrosis	8 (11.9)	59 (88.1)
Emphysema	8 (11.9)	59 (88.1)
Bullae	2 (3.0)	65 (97.0)

Finally, a multidisciplinary discussion involving three pulmonologists and one pathologist was conducted to assess the possibility of IPF, based on imaging and histopathological features, and thus establish the clinical diagnosis through integration of all available clinical information. HRCT images and histopathological specimens were reviewed in separate sessions using de-identified material, so that a given image could not be linked to the corresponding specimen or patient, even though one evaluator (S.H.) participated in both assessments.

### Statistical analysis

Data are presented as medians with interquartile ranges, or as frequencies and percentages, as appropriate. Categorical variables were compared using the Pearson chi-square test or Fisher’s exact test. Continuous variables were compared using the Mann–Whitney U test, or the Kruskal–Wallis test for comparisons involving more than two groups. All *p*-values were two-sided, and a *p*-value < 0.05 was considered statistically significant. To account for multiple comparisons across the analysis of 24 histopathological features, false discovery rate correction was applied using the step-up method of Benjamini, Krieger, and Yekutieli; statistical significance was defined as *q* < 0.05. For univariate analyses used for variable screening prior to multivariate modelling, uncorrected *p*-values were applied. Logistic regression models were constructed to identify factors associated with BALF lymphocyte proportion. Three variables were entered into the multivariable model, selected as those with the smallest p values in univariate analysis, in accordance with a limit of 10 events per variable. Odds ratios, 95% confidence intervals, and p values were derived from profile likelihood. Multicollinearity was assessed using pairwise phi coefficients and variance inflation factors. Firth penalized maximum likelihood estimation was applied as a sensitivity analysis, in which all five variables significant on univariate testing were entered simultaneously. BALF lymphocytosis was defined by a threshold of 15%, taken from the 2012 American Thoracic Society clinical practice guideline, yielding groups of 38 and 29 patients. A threshold of 18.5%, derived from receiver operating characteristic (ROC) analysis in the present cohort, was used to describe the discrimination of fibroblastic foci and yields groups of 36 and 31 patients; a multivariable model using this threshold is reported as a sensitivity analysis. The event of interest was death from any cause. Survival time was measured from the date of SLB to death or to the date of last confirmed follow-up, and the median duration of follow-up was estimated using the reverse Kaplan–Meier method. To assess whether BALF cellular fractions add to radiological information, logistic regression models for the presence of fibroblastic foci were fitted with the HRCT pattern alone, the BALF lymphocyte proportion alone, or both, and areas under the ROC curve were compared using the DeLong test with bootstrap optimism correction (2000 replications). These analyses, together with the stratified, adjusted, and sensitivity analyses described above, were exploratory and were not prespecified. Statistical analyses were conducted using GraphPad Prism version 10 (GraphPad Software, San Diego, CA), IBM SPSS Statistics version 22 (IBM Corp., Armonk, NY), and R version 4.5.2 (R Foundation for Statistical Computing, Vienna, Austria) with the logistf package for Firth penalized estimation and the pROC package for comparison of ROC curves.

## Results

### Baseline characteristics and pathological features of the patients

The baseline demographics of the study population are summarized in Table 1. A total of 67 patients with IP were included; 34 were male and 33 were female, with a median age of 64 years. None of the patients were receiving treatment for IP, including immunosuppressive therapy, at the time of the BALF collection. The median BALF recovery rate was 53% (interquartile range [IQR], 47–61%). Median differential cell proportions were as follows: macrophages 68% (IQR, 34–79%), neutrophils 4% (IQR, 1–7%), lymphocytes, 21% (IQR, 8–34%), and eosinophils 3% (IQR, 1–6%). Based on HRCT, IP patterns were probable UIP in 10 patients, indeterminate for UIP in 12, and alternative diagnosis in 45; no patients met the criteria for definite UIP on HRCT. Histopathological patterns were classified as UIP in 2 patients, probable UIP in 9, indeterminate for UIP in 12, and alternative diagnosis in 44. Final diagnoses included IPF in 9 patients, NSIP in 19, unclassifiable IP in 23, CTD-ILD in 9, and chronic hypersensitivity pneumonitis (CHP) in 7.

The pathological features of the study population are summarized in Table 2. The most frequently observed pathological features were alveolitis and fibrosis (each n = 62, 92.5%), whereas bullae were the least frequent (n = 2, 3.0%), followed by pleural disease and vascular endothelial hypertrophy (each n = 3, 4.5%).

### BALF lymphocyte proportion in relation to histopathological and CT findings

First, BALF lymphocyte proportion was compared with lung histopathology obtained from SLB. Fibroblastic foci demonstrated significant negative association with BALF lymphocyte proportion (median, 13.0% vs. 29.0%; *q =* 0.002). Conversely, positive associations were observed for bronchiolitis (median, 29.0% vs. 14.0%; *q* = 0.029), intraluminal organization (median, 48.0% vs. 14.0%; *q* = 0.002), and epithelioid cell granulomas (median, 71.5% vs. 18.0%; *q* = 0.012) (Fig 1a–d, S1 Table).

**Fig 1:**
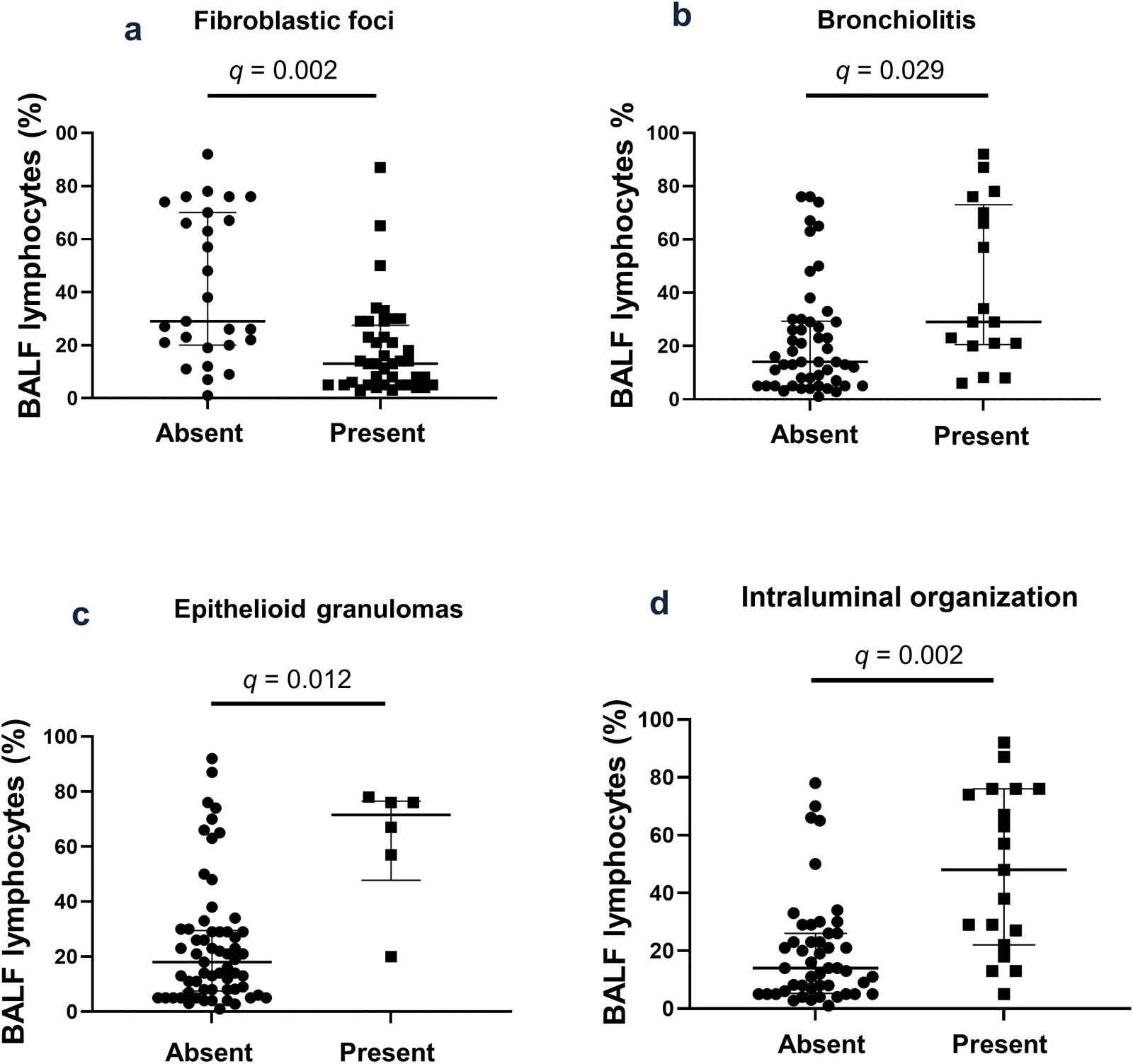
BALF lymphocyte proportion according to histopathological features. (a-d) Box plots comparing BALF lymphocyte proportion in patients with versus without fibroblastic foci, bronchiolitis, epithelioid cell granuloma, and intraluminal organization. *q*-values represent false discovery rate-adjusted *p*-values. BALF, bronchoalveolar lavage.

When stratified by histopathological pattern, BALF lymphocyte proportion was significantly lower in patients with UIP or probable UIP than in those with indeterminate for UIP or alternative diagnosis patterns (median, 6.0% vs. 22.5%; *p* = 0.023) (Fig 2a). Similarly, per HRCT pattern, BALF lymphocyte proportion was lower in probable UIP than in the alternative diagnosis group (median, 5.5% vs. 21.0%; *p* = 0.045) (Fig 2b).

**Fig 2:**
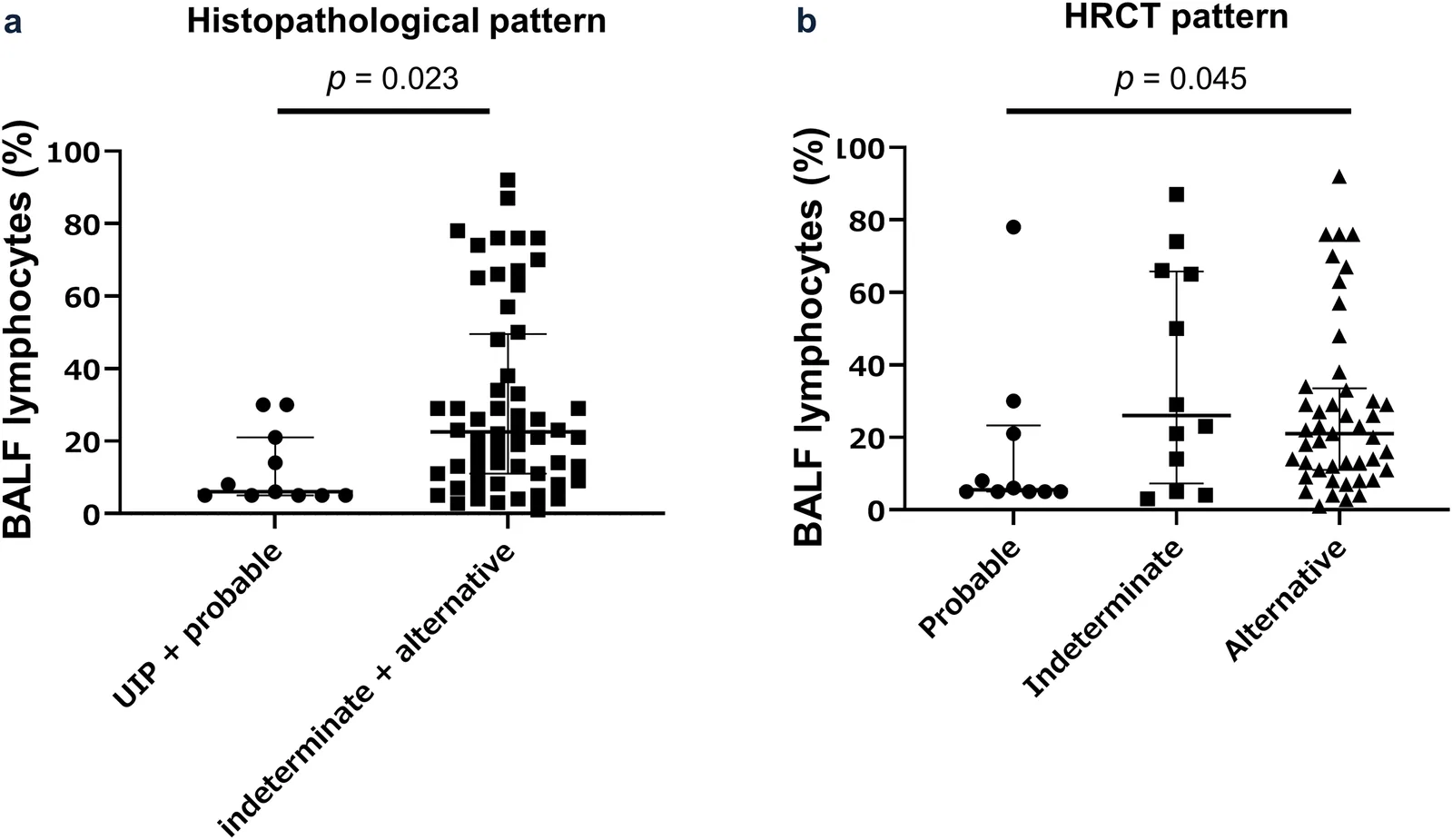
BALF lymphocyte proportion by pathological and HRCT patterns. (a) Pathological patterns: UIP/probable UIP (n = 11) versus indeterminate/alternative diagnosis (n = 56). (b) HRCT patterns: probable UIP (n = 10) versus indeterminate (n = 12) versus alternative diagnosis (n = 45). p-values were calculated using the Mann–Whitney U test. BALF, bronchoalveolar lavage; UIP, usual interstitial pneumonia; HRCT, high-resolution computed tomography.

Fibroblastic foci remained associated with a lower BALF lymphocyte proportion after excluding patients with a UIP or probable UIP pattern (14.0% vs 29.0%, n = 56, p = 0.002) and after excluding patients with IPF (13.0% vs 29.0%, n = 58, p = 0.001). This association was also observed among the 19 patients with idiopathic NSIP (13.0% vs 32.5%, p = 0.006), whereas a similar trend was observed among the 23 patients with unclassifiable IP, although the difference did not reach statistical significance (p = 0.067). The results of all subgroup analyses are presented in Table 4, Panel A.

BALF lymphocyte proportion provided additional discriminatory information beyond the radiological pattern. In logistic regression models for the presence of fibroblastic foci, the AUC was 0.686 for the HRCT pattern alone, 0.764 for the BALF lymphocyte proportion alone, and 0.847 for the combined model. The combined model had a significantly higher AUC than the HRCT pattern alone (DeLong p = 0.005), with a bootstrap-corrected AUC of 0.827.

### Multivariable analysis of factors associated with BALF lymphocytosis

In accordance with the 2012 American Thoracic Society clinical practice guideline [6], a BALF lymphocyte proportion of ≥ 15% was used to define BALF lymphocytosis, and to assess associations with histopathological features. In univariate analyses, BALF lymphocytosis was significantly associated with the presence of fibroblastic foci [42.1% (16/38) vs. 82.8% (24/29); *p* = 0.001], bronchiolitis [36.8% (14/38) vs. 10.3% (3/29); *p* = 0.022], intraluminal organization [42.1% (16/38) vs. 10.3% (3/29); *p* = 0.006], epithelioid cell granulomas [15.8% (6/38) vs. 0% (0/29); *p* = 0.032], and intraluminal lymphocytic proliferation [36.8% (14/38) vs. 6.9% (2/29); *p* = 0.008] (Table 3).

**Table 3:** Univariate and multivariable analysis of factors associated with BALF lymphocytosis (≥15%).

Variable(s)	BALF Lymphocytosis (*n* = 38)	BALF Non-Lymphocytosis (*n* = 29)	Univariate	Multivariate		
			*p* value	*p* value	Odds ratio	95% CI
Age, > 65 years	19 (50.0)	12 (41.4)	0.623			
Sex, Male	16 (42.1)	18 (62.1)	0.141			
Smoking history, Never	21 (55.3)	11 (37.9)	0.218			
Pathological features, (present)						
Alveolitis	37 (97.4)	25 (86.2)	0.174			
Fibrosis	33 (86.8)	29 (100)	0.064			
Fibroblastic foci	16 (42.1)	24 (82.8)	0.001	0.018	0.23	0.06–0.78
Subpleural and perilobular distribution	19 (50.0)	18 (62.1)	0.461			
Bronchiolitis	14 (36.8)	3 (10.3)	0.022			
Intraluminal organization	16 (42.1)	3 (10.3)	0.006	0.099	3.36	0.80–17.38
Bronchiectasis	30 (79.0)	26 (89.7)	0.326			
Honeycomb	3 (7.9)	3 (10.3)	>0.999			
Epithelioid cell granuloma	6 (15.8)	0 (0.0)	0.032			
Bronchovascular bundle thickening	30 (79.0)	22 (75.9)	0.776			
Lymphoid hyperplasia	34 (89.5)	22 (75.9)	0.187			
Lymphoid follicle	14 (36.8)	14 (48.3)	0.454			
Vascular Endothelial Hypertrophy	0 (0.0)	3 (10.3)	0.076			
Intraluminal macrophage proliferation	5 (13.2)	2 (6.9)	0.690			
Intraluminal neutrophil proliferation	8 (21.1)	2 (6.9)	0.168			
Intraluminal eosinophil proliferation	3 (7.9)	3 (10.3)	>0.999			
Intraluminal lymphocyte proliferation	14 (36.8)	2 (6.9)	0.008	0.035	5.25	1.12–38.21
Intraluminal plasma cell proliferation	6 (15.8)	1 (3.4)	0.129			
Type Ⅱ pneumocyte hyperplasia	35 (92.1)	26 (89.7)	>0.999			
Bronchial epithelial cell hyperplasia	33 (86.8)	26 (89.7)	>0.999			
Pleural disease	3 (7.9)	0 (0.0)	0.252			
Airspace enlargement with fibrosis	3 (7.9)	5 (17.2)	0.278			
Emphysema	4 (10.5)	4 (13.8)	0.719			
Bullae	1 (2.6)	1 (3.4)	>0.999			

Data are presented as *n* (%). Multivariable analysis was performed using logistic regression. Blank cells denote variables that were not entered into the multivariable model. BALF, bronchoalveolar lavage fluid.

In the multivariable logistic regression analysis, fibroblastic foci were independently associated with a lower likelihood of BALF lymphocytosis (odds ratio [OR] 0.23, 95% CI 0.06–0.78, p = 0.018). Intraluminal lymphocyte proliferation was associated with a higher likelihood of BALF lymphocytosis (OR 5.25, 95% CI 1.12–38.21, p = 0.035), based on 14 patients with the feature among those with lymphocytosis and 2 among those without (Table 3).

In a Firth penalized model containing all five variables significant on univariate testing, fibroblastic foci retained a significant association (OR 0.29, 95% CI 0.08–0.97, p = 0.045), whereas the remaining estimates were unstable (S4 Table).

The association between fibroblastic foci and BALF lymphocytosis was retained when the histopathological pattern was added to the model (OR 0.27, 95% CI 0.07–0.95, p = 0.041) and when a final diagnosis of IPF was added (OR 0.22, 95% CI 0.06–0.79, p = 0.019), while neither the pattern term nor the diagnosis term was itself significant (Table 4, Panel B).

**Table 4:** Association between fibroblastic foci and BALF lymphocyte proportion in stratified and adjusted analyses.

Panel A. BALF lymphocyte proportion according to the presence of fibroblastic foci				
Analysis population	n	Fibroblastic foci present	Fibroblastic foci absent	*p* value
All patients	67	40; 13.0 (5.0–24.5)	27; 29.0 (20.5–68.5)	<0.001
***Stratified by histopathological pattern***				
Excluding UIP and probable UIP	56	29; 14.0 (8.0–29.0)	27; 29.0 (20.5–68.5)	0.002
Alternative diagnosis pattern only	44	19; 13.0 (8.1–22.0)	25; 27.0 (20.0–67.0)	0.007
***Stratified by final multidisciplinary diagnosis***				
Excluding IPF	58	31; 13.0 (6.5–29.0)	27; 29.0 (20.5–68.5)	0.001
Idiopathic NSIP only	19	9; 13.0 (8.0–18.0)	10; 32.5 (22.2–59.2)	0.006
Unclassifiable IP only	23	19; 13.0 (5.0–29.5)	4; 47.5 (26.8–72.5)	0.067
**Panel B. Multivariable models additionally adjusted for disease category**				
**Variable**		**Odds ratio**	**95% CI**	***p* value**
***Model 1: adjusted for histopathological pattern***				
Fibroblastic foci		0.27	0.07–0.95	0.041
Intraluminal organization		3.18	0.77–16.42	0.113
Intraluminal lymphocyte proliferation		4.96	1.07–36.01	0.040
UIP or probable UIP pattern		0.55	0.09–2.64	0.462
***Model 2: adjusted for a final diagnosis of IPF***				
Fibroblastic foci		0.22	0.06–0.79	0.019
Intraluminal organization		3.43	0.81–17.98	0.095
Intraluminal lymphocyte proliferation		5.18	1.10–37.81	0.037
Final diagnosis of IPF		1.24	0.22–6.36	0.798

Panel A shows the number of patients and the median (interquartile range) BALF lymphocyte proportion, expressed as a percentage. The first block stratifies patients by the histopathological pattern assigned to the surgical lung biopsy specimen, and the second by the final diagnosis established through multidisciplinary discussion.

In Panel B, the three histopathological variables of the primary model were entered together with an indicator of disease category. Because fibroblastic foci contribute to the definition of the UIP pattern, Model 1 carries a risk of overadjustment and is presented as a supportive analysis alongside Panel A.

BALF, bronchoalveolar lavage fluid; CI, confidence interval; IPF, idiopathic pulmonary fibrosis; NSIP, nonspecific interstitial pneumonia; IP, interstitial pneumonia; UIP, usual interstitial pneumonia.

In a sensitivity analysis using the 18.5% threshold as the outcome, fibroblastic foci remained associated with a lower likelihood of lymphocytosis (OR 0.17, 95% CI 0.04–0.58, p = 0.004) and intraluminal lymphocyte proliferation with a higher likelihood (OR 6.60, 95% CI 1.40–48.50, p = 0.016), consistent in direction with the primary analysis (S5 Table).

### Survival analysis according to histopathological features and BALF lymphocytosis

Kaplan–Meier survival analysis demonstrated a significantly worse prognosis in patients with fibroblastic foci compared with those without fibroblastic foci (log-rank test, *p* = 0.003), whereas no significant survival difference was observed with intraluminal lymphocytic proliferation (log-rank test, *p* = 0.080) (Fig 3a and 3b). Nineteen of the 67 patients died during follow-up, comprising 16 of the 40 patients with fibroblastic foci (40.0%) and 3 of the 27 patients without them (11.1%). Median survival was 8.3 years in the presence of fibroblastic foci and was not reached in their absence. The median duration of follow-up estimated by the reverse Kaplan–Meier method was 7.5 years, with observed follow-up ranging from 0.2 to 15.9 years. No patient underwent lung transplantation during follow-up.

**Fig 3:**
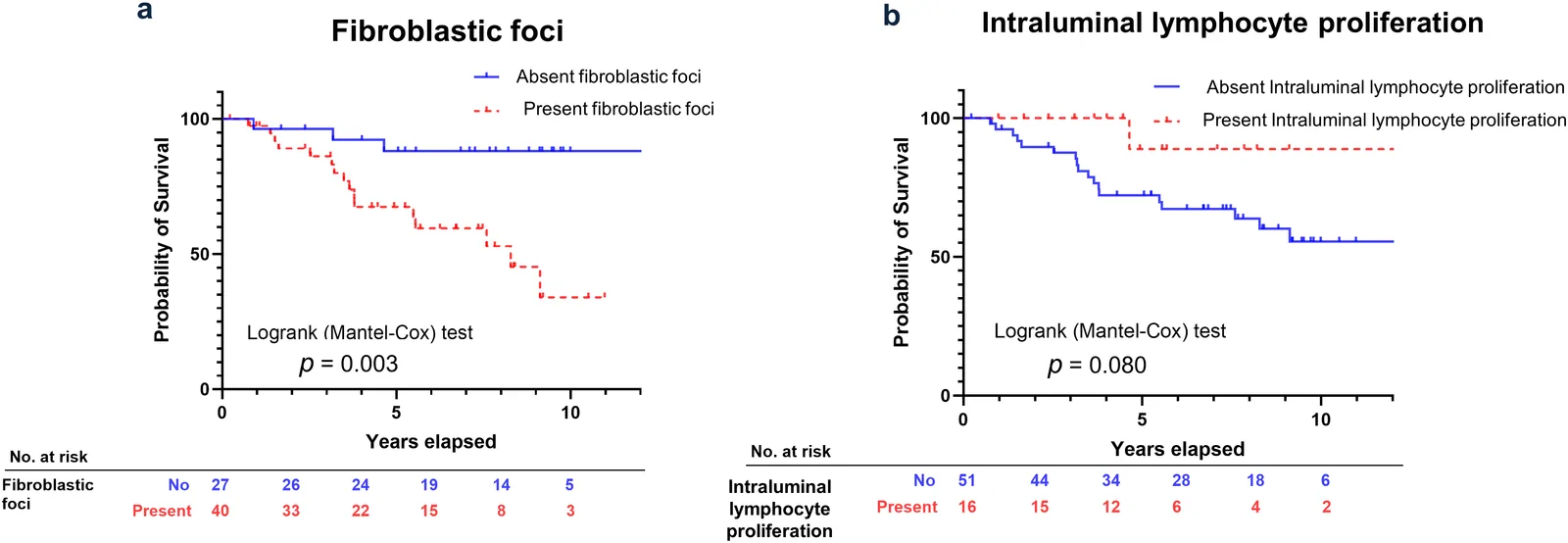
Survival analysis. (a) Kaplan–Meier survival curves stratified by the presence (red dashed line) or absence (blue solid line) of fibroblastic foci. Survival was significantly shorter in patients with fibroblastic foci (*p* = 0.003). (b) Kaplan–Meier survival curves stratified by the presence (red dashed line) or absence (blue solid line) of intraluminal lymphocyte proliferation. No significant difference in survival was observed (*p* = 0.080). P-values for survival analyses were calculated using the log-rank (Mantel–Cox) test. Numbers at risk shown below. BALF, bronchoalveolar lavage fluid.

Given the prognostic significance of fibroblastic foci, ROC curve analysis was performed to determine the optimal BALF lymphocyte proportion for predicting their presence. The optimal cutoff value was 18.5% (AUC 0.763, 95% CI: 0.646–0.882, sensitivity 65.0%, specificity 81.5%) (S1 Fig).

No statistically significant difference in overall survival was observed between patients with BALF lymphocytosis (≥18.5%) and those without lymphocytosis (<18.5%) (log-rank test, *p* = 0.104) (S2 Fig). The proportion of patients with IPF did not differ significantly between the lymphocytosis group (3/36, 8.3%) and the non-lymphocytosis group (6/31, 19.4%) (*p* = 0.284). Similarly, corticosteroid use after BALF collection and SLB did not differ significantly between groups (63.9% vs 48.4%, *p* = 0.226). The distribution of non-IPF subtypes was comparable between the two groups. No significant difference in overall survival was observed when the guideline threshold of 15% was applied instead (p = 0.413).

### Association of BALF fluids neutrophil or eosinophil proportions with histopathological features

Next, neutrophil and eosinophil proportions in BALF were compared with lung histopathological features obtained from SLB specimens. No histopathological features showed a significant association with neutrophil proportion in BALF (S2 Table). In contrast, vascular endothelial hypertrophy was positively associated with the BALF eosinophil proportion (median, 21.0% vs. 3.0%; q = 0.038), although this feature was present in only 3 patients (S3 Table; S3 Fig).

### BALF cellular fractions in relation to the final diagnosis

BALF lymphocyte proportion did not differ significantly among the five final diagnoses (p = 0.306). Median values were 6.0% in IPF, 21.0% in idiopathic NSIP, 20.0% in unclassifiable IP, 22.0% in CTD-ILD, and 67.0% in CHP, and the number of patients in each category was small. When IPF was compared with the other diagnoses combined, the difference was in the expected direction but did not reach significance (6.0% versus 22.0%, p = 0.068), and discrimination was limited (AUC 0.691). Among the 57 patients whose HRCT pattern was not probable UIP, only 3 had IPF, and discrimination was lower still (AUC 0.657, Mann–Whitney p = 0.371).

The radiological pattern and the final diagnosis were discordant in 7 patients. Among the 4 patients with a probable UIP pattern in whom the final diagnosis was not IPF, BALF lymphocyte proportions were 5.0%, 8.0%, 5.0%, and 78.0%; the three lower values were indistinguishable from the median of 5.5% observed in the 6 patients with a probable UIP pattern and a final diagnosis of IPF, whereas the single patient with marked lymphocytosis received a final diagnosis of CHP and had epithelioid cell granulomas on histology. Among the 3 patients with IPF whose HRCT pattern was not probable UIP, BALF lymphocyte proportions were 5.0%, 16.0%, and 23.0%. These numbers are too small to support inference.

## Discussion

The purpose of this study was to evaluate the associations between distinctive lung histopathological features and BALF cellular fractions in patients with IP. Histopathological features of IP are heterogenous and frequently coexist, making it challenging to infer underlying pathology without invasive procedures. Clarifying how specific histopathological features are reflected in BALF cellular composition may therefore enhance the utility of BALF as a non-invasive adjunctive tool for pathological assessment in IP.

In our study, we observed a significant negative association between BALF lymphocyte proportion and pulmonary fibroblastic foci. On multivariable analysis, fibroblastic foci were independently associated with a low BALF lymphocyte proportion (OR 0.23, 95% CI 0.06–0.78). Intraluminal lymphocyte proliferation showed a similar association in the opposite direction, but the estimate rested on few patients and should be regarded as exploratory (OR 5.25, 95% CI 1.12–38.21). Importantly, fibroblastic foci were strongly associated with poorer survival, consistent with their established prognostic significance in IP. Taken together, these results indicate that BALF cellular fractions are associated with individual histopathological features, particularly the presence of fibroblastic foci, and may therefore provide complementary information when histological material is not available. Recognition of fibroblastic foci is clinically relevant, because this feature is among the most prognostically informative histopathological findings in patients with IP.

To our knowledge, no prior studies have systematically compared BALF cellular fractions with specific lung histopathological features. Our study identified a significant negative association between BALF lymphocyte proportion and the presence of fibroblastic foci, a key histopathological feature of UIP. Several previous studies have reported that fibroblastic foci are strongly associated with poor prognosis in patients with IP [15]. In both UIP and NSIP, a higher number or greater extent of fibroblastic foci has been linked to worse clinical outcomes [16]. Similarly, in CHP, the burden of fibroblastic foci correlates significantly with prognosis [17,18]. In unclassifiable IP, rapidly progressive disease is characterized by a higher proportion of fibroblastic foci and poorer survival compared with slowly progressive cases [19]. Moreover, in primary Sjögren’s syndrome associated–ILD, the severity of fibroblastic foci has also been shown to correlate with prognosis [20]. Collectively, these findings underscore fibroblastic foci as a shared and robust prognostic factor across fibrotic and chronic ILDs, irrespective of etiology or radiologic pattern. Recognising the presence of fibroblastic foci is therefore relevant to prognostic assessment in ILD, and our findings suggest that BALF cellular analysis may contribute complementary information towards that assessment.

Fibroblastic foci are a defining feature of the UIP pattern, and a low BALF lymphocyte proportion is well recognised in both UIP and IPF. Our finding could therefore simply reflect this established association. However, the distribution of fibroblastic foci in our cohort suggests that this explanation is insufficient. Of the 40 patients with fibroblastic foci, 29 had a histopathological pattern other than UIP or probable UIP, and 31 had a final diagnosis other than IPF. The association was also maintained in the stratified analyses, including among patients with alternative histopathological patterns and those with idiopathic NSIP. It remained significant in models adjusted separately for histopathological pattern and for a final diagnosis of IPF, although these adjustments may result in overadjustment because of the definitional overlap. The stratified analyses are therefore the more informative comparison, and they suggest that the association is unlikely to be explained by the UIP pattern or by a diagnosis of IPF alone.

The anatomical origin of BALF recovery remains incompletely understood; it is unclear whether BALF predominantly derives from and samples peripheral alveolar regions or more central bronchiolar airspaces. Because the specific sampling compartment has not been directly established it remains uncertain whether BALF findings accurately reflect alveolar or interstitial pathology. Our results partially support this uncertainty: BALF lymphocyte proportions showed no association with interstitial pathological features (alveolitis, lymph follicles, lymphoid hyperplasia). In contrast, multivariable analysis demonstrated a significant association between intraluminal lymphocytic infiltration and BALF lymphocyte proportion. Together, these findings suggest a critical distinction—BALF may not accurately reflect cellular proliferation within interstitial lesions but rather preferentially samples cells present within the alveolar space. Thus, BALF composition may more closely reflect cellular content within the alveolar lumen than within the interstitium. This may explain why BALF correlates with intraluminal but not interstitial lymphocytic infiltration.

The relationship between BALF lymphocyte proportion and prognosis in IP remains controversial. While some studies have reported that BALF lymphocytosis is associated more with a better prognosis in patients with UIP [21], others have found no significant association between BALF lymphocytosis and survival [11,13]. In our cohort, we found no significant difference in survival between patients with BALF lymphocyte proportion ≥ 18.5% and those with lower values. This lack of statistical significance may be attributable to several factors, including the relatively small sample size (n = 67), which may have limited statistical power, as well as the heterogeneity of IP subtypes included in our study. In contrast to studies focusing exclusively on UIP, our cohort comprised multiple IP subtypes, which may have contributed to the diluted results. Furthermore, although we adopted a 15% cutoff for BALF lymphocytosis in multivariable analysis based on ATS guidelines, the appropriateness of this threshold for a heterogeneous IP cohort remains uncertain. Given the strong negative association between BALF lymphocyte proportion and fibroblastic foci in our study, and the well-established prognostic significance of fibroblastic foci, larger multicenter studies will be required to further clarify the prognostic value of BALF lymphocytosis in IP.

The contrast between the two survival analyses merits attention. Stratification by the histological feature itself clearly separated the survival curves, whereas stratification by BALF lymphocyte proportion did not, either at the 18.5% threshold (p = 0.104) or at the guideline threshold of 15% (p = 0.413). The ability of BALF lymphocyte proportion to discriminate fibroblastic foci was moderate, with an AUC of 0.763 and a sensitivity of 65.0% at the 18.5% threshold, indicating that approximately one third of patients with fibroblastic foci would not be identified. Taken together, these observations suggest that BALF cellular analysis provides only partial information on the presence of fibroblastic foci and is unlikely to substitute for histological information in prognostic assessment.

These observations may also indicate where the information carried by BALF is most readily found. BALF lymphocyte proportion did not distinguish among the five final diagnoses in this cohort, nor did it resolve cases in which the HRCT pattern and final diagnosis disagreed. In contrast, it was associated with fibroblastic foci across the diagnostic categories examined. Previous studies have compared BALF findings with histopathological patterns or final diagnoses, and our results raise the possibility that specific histological features may provide a more informative comparator. This observation is nonetheless based on a single histological feature in a single cohort and requires confirmation in independent studies.

The observed association between pulmonary fibroblastic foci and BALF lymphocyte proportion suggests that BALF cellular composition may indeed reflect alveolar pathology indirectly, rather than directly via sampling of alveolar regions. Previous studies have demonstrated that fibroblasts in patients with IP can suppress T-cell proliferation, induce T-cell apoptosis, and inhibit lymphocyte migration [22]. These findings raise the possibility that pathological fibroblast proliferation within the lung may contribute to lymphocyte depletion in BALF. Conversely, T lymphocytes have been shown to play a role in fibroblast clearance [23], suggesting plausibly that reduced T lymphocyte numbers could impair the removal of fibroblasts, thereby promote persistence or progression of abnormal fibroblastic foci proliferation. The negative association observed in our study between BALF lymphocyte proportion and pulmonary fibroblastic foci is consistent with these previous reports. However, the directionality and causality of this relationship, whether reduced lymphocyte numbers contribute to the formation of fibroblastic foci or the reverse, remain to be ascertained.

Several limitations warrant consideration. First, this was a single-center retrospective study of a relatively small cohort composed predominantly of patients who required SLB, so selection bias cannot be excluded and the findings may not be generalizable; the analyses in Table 4 and the supplementary tables were exploratory rather than prespecified, and the models were developed and evaluated in the same cohort without external validation. Second, BAL was performed in different lung segments according to disease distribution, and the two procedures were separated by a median of 43 days, so the two specimens may not represent the same disease state. No patient was receiving treatment for interstitial pneumonia at the time of BAL and patients with documented progression between the procedures were excluded, but change during the interval cannot be excluded. Third, histopathological features were recorded as present or absent, so associations with the extent of each feature could not be examined; several features were observed in very few patients, and associations involving vascular endothelial hypertrophy, pleural disease, bullae, and epithelioid cell granulomas should be regarded as hypothesis generating. One evaluator also participated in the radiological assessment, the histopathological assessment, and the multidisciplinary discussion, and although the radiological and histopathological reviews were conducted separately on de-identified material, complete independence of assessment cannot be guaranteed. Finally, treatment was not standardised and serial pulmonary function data were not collected, so the relationship between BALF cellular fractions and treatment response could not be examined. Further prospective multicenter studies are warranted to validate these findings and clarify the clinical utility of BALF cellular analysis as a complementary tool for histopathological assessment in IP.

## Conclusions

In conclusion, this study demonstrates that BALF cellular fractions are associated with specific histopathological features in IP, most notably a negative association between BALF lymphocyte proportion and fibroblastic foci that persisted across diagnostic categories. Given that fibroblastic foci have prognostic significance across multiple ILD subtypes, BALF cellular analysis may provide complementary information on histopathological features when surgical lung biopsy is not feasible. However, the moderate discriminatory performance observed in this study indicates that BALF is unlikely to substitute for histological assessment.

## Supporting information

S1 Dataset De-identified patient-level dataset. Clinical, bronchoalveolar lavage fluid, and histopathological variables for the 67 patients analyzed in this study. (XLSX)

S1 Fig ROC curve for predicting fibroblastic foci by BALF lymphocyte proportion. Receiver operating characteristic (ROC) curve analysis of BALF lymphocyte proportion for predicting the presence of fibroblastic foci. The optimal cutoff value was 18.5% (AUC 0.763, 95% CI 0.646–0.882, sensitivity 65.0%, specificity 81.5%). BALF, bronchoalveolar lavage fluid; AUC, area under the curve; CI, confidence interval.
(TIF)

S2 Fig Survival analysis stratified by BALF lymphocytosis. Kaplan–Meier survival curves stratified by BALF lymphocyte proportion using the ROC-derived cutoff of 18.5%. The red dashed line represents patients without BALF lymphocytosis (<18.5%) and the blue solid line represents patients with lymphocytosis (≥18.5%). No statistically significant difference in overall survival was observed between the two groups (log-rank test, p = 0.104). BALF, bronchoalveolar lavage fluid.
(TIF)

S3 Fig BALF eosinophil proportion and vascular endothelial hypertrophy. Box plot comparing BALF eosinophil proportion in patients with versus without vascular endothelial hypertrophy. The q-value represents the false discovery rate-adjusted p-value. BALF, bronchoalveolar lavage fluid.
(TIF)

S1 Table Comparison of BALF lymphocyte proportion according to the presence or absence of pathological features. (DOCX)

S2 Table Comparison of BALF neutrophil proportion according to histopathological features. (DOCX)

S3 Table Comparison of BALF eosinophil proportion according to the presence or absence of histopathological features. (DOCX)

S4 Table Sensitivity analysis using Firth penalized maximum likelihood estimation. (DOCX)

S5 Table Multivariable analysis using two definitions of BALF lymphocytosis. (DOCX)
