# Supplementary material for: Bronchoalveolar lavage fluid cellular fractions reflect individual histopathological features in interstitial pneumonia
Source: PLoS One. 2026 Oct 30;21(10):e0361768. doi: 10.1371/journal.pone.0361768 (PMC13722173; doi:10.1371/journal.pone.0361768)
Supplement: Supplementary file 1 [file pone.0361768.s005.docx]

**S1 Table. Comparison of BALF lymphocyte proportion according to the presence or absence of pathological features**

| **Histopathological feature** | **n (HF+)** | **BALF Lymphocytes, % (HF+)** | **n (HF–)** | **BALF Lymphocytes, % (HF–)** | **p value** | **q value** |
| --- | --- | --- | --- | --- | --- | --- |
| Alveolitis | 62 | 21.0 (8.0–35.0) | 5 | 13.0 (5.0–35.5) | 0.330 | 0.597 |
| Fibrosis | 62 | 18.5 (7.8–30.8) | 5 | 38.0 (21.0–74.5) | 0.078 | 0.234 |
| Fibroblastic foci | 40 | 13.0 (5.0–27.5) | 27 | 29.0 (20.0–70.0) | <0.001 | 0.002 |
| Subpleural and perilobular distribution | 37 | 16.0 (5.0–33.5) | 30 | 21.5 (11.8–40.5) | 0.512 | 0.597 |
| Bronchiolitis | 17 | 29.0 (20.5–73.0) | 50 | 14.0 (5.0–29.3) | 0.005 | 0.029 |
| Intraluminal organization | 19 | 48.0 (22.0–78.0) | 48 | 14.0 (5.3–26.0) | <0.001 | 0.002 |
| Bronchiectasis | 56 | 18.5 (8.0–32.3) | 11 | 26.0 (9.0–63.0) | 0.405 | 0.597 |
| Honeycombing | 6 | 14.5 (5.8–68.3) | 61 | 21.0 (8.1–33.5) | 0.902 | 0.824 |
| Epithelioid cell granulomas | 6 | 71.5 (47.8–76.5) | 61 | 18.0 (7.5–29.5) | 0.002 | 0.012 |
| Bronchovascular bundle thickening | 52 | 21.0 (8.1–32.3) | 15 | 16.5 (5.0–50.0) | 0.867 | 0.824 |
| Lymphoid hyperplasia | 56 | 21.5 (8.4–33.8) | 11 | 13.0 (5.0–67.0) | 0.434 | 0.597 |
| Lymphoid follicles | 28 | 16.5 (8.0–28.3) | 39 | 22.5 (8.0–57.0) | 0.253 | 0.586 |
| Vascular endothelial hypertrophy | 3 | 9.0 (8.0–14.0) | 64 | 21.0 (8.0–37.0) | 0.279 | 0.586 |
| Intraluminal macrophage proliferation | 7 | 48.0 (9.0–76.0) | 60 | 20.0 (8.0–30.0) | 0.210 | 0.552 |
| Intraluminal neutrophil proliferation | 10 | 22.0 (15.5–66.3) | 57 | 19.0 (7.5–33.5) | 0.457 | 0.597 |
| Intraluminal eosinophil proliferation | 6 | 18.5 (12.0–23.0) | 61 | 21.0 (7.5–43.0) | 0.769 | 0.769 |
| Intraluminal lymphocyte proliferation | 16 | 26.0 (21.0–60.0) | 51 | 14.0 (7.0–30.0) | 0.041 | 0.172 |
| Intraluminal plasma cell proliferation | 7 | 22.0 (21.0–48.0) | 60 | 18.5 (8.0–33.8) | 0.497 | 0.597 |
| Type II pneumocyte hyperplasia | 61 | 21.0 (8.0–31.5) | 6 | 27.0 (11.0–61.8) | 0.509 | 0.597 |
| Bronchial epithelial cell hyperplasia | 59 | 20.0 (8.0–34.0) | 8 | 25.5 (7.0–0.0) | 0.585 | 0.614 |
| Pleural disease | 3 | 76.0 (20.0–78.0) | 64 | 20.0 (8.0–32.3) | 0.076 | 0.234 |
| Airspace enlargement with fibrosis | 8 | 11.5 (5.8–56.0) | 59 | 21.0 (8.0–34.0) | 0.565 | 0.614 |
| Emphysema | 8 | 23.5 (5.8–61.3) | 59 | 21.0 (8.0–30.0) | 0.974 | 0.852 |
| Bullae | 2 | 13.5 (4.0–23.0) | 65 | 21.0 (8.0–36.0) | 0.411 | 0.597 |

Data are presented as median (interquartile range). False discovery rate (FDR) correction was applied using the Benjamini–Krieger–Yekutieli step-up method, with q < 0.05 considered statistically significant. BALF, bronchoalveolar lavage fluid; HF+, histopathological features present; HF–, histopathological features absent.
