# Supplementary material for: Bronchoalveolar lavage fluid cellular fractions reflect individual histopathological features in interstitial pneumonia
Source: PLoS One. 2026 Oct 30;21(10):e0361768. doi: 10.1371/journal.pone.0361768 (PMC13722173; doi:10.1371/journal.pone.0361768)
Supplement: Supplementary file 2 [file pone.0361768.s006.docx]

**S2 Table. Comparison of BALF neutrophil proportion according to histopathological features**

| **Histopathological feature** | **n (HF+)** | **BALF Neutrophils, % (HF+)** | **n (HF–)** | **BALF Neutrophils, % (HF–)** | **p value** | **q value** |
| --- | --- | --- | --- | --- | --- | --- |
| Alveolitis | 62 | 3.5 (1.0–7.5) | 5 | 4.0 (1.5–16.0) | 0.911 | >0.999 |
| Fibrosis | 62 | 3.5 (1.0–7.0) | 5 | 4.0 (0.5–33.0) | 0.866 | >0.999 |
| Fibroblastic foci | 40 | 4.0 (1.0–9.3) | 27 | 3.0 (1.0–7.0) | 0.517 | 0.941 |
| UIP pattern | 37 | 4.0 (1.5–6.9) | 30 | 3.5 (1.0–12.0) | 0.927 | >0.999 |
| Bronchiolitis | 17 | 6.8 (1.5–21.5) | 50 | 3.0 (1.0–6.0) | 0.219 | 0.690 |
| Intraluminal organization | 19 | 2.0 (1.0–4.0) | 48 | 4.0 (2.0–10.0) | 0.026 | 0.295 |
| Bronchiectasis | 56 | 4.0 (1.0–9.8) | 11 | 3.0 (0.0–4.0) | 0.156 | 0.562 |
| Honeycombing | 6 | 10.0 (1.0–28.0) | 61 | 4.0 (1.0–7.0) | 0.632 | >0.999 |
| Epithelioid cell granuloma | 6 | 5.0 (0.8–29.5) | 61 | 3.0 (1.0–7.0) | 0.694 | >0.999 |
| Bronchovascular bundle thickening | 52 | 4.0 (1.3–12.3) | 15 | 3.0 (0.0–4.0) | 0.022 | 0.295 |
| Lymphoid hyperplasia | 56 | 3.5 (1.3–8.5) | 11 | 4.0 (1.0–6.0) | 0.753 | >0.999 |
| Lymphoid follicle | 28 | 3.0 (1.3–8.5) | 39 | 4.0 (1.0–7.0) | 0.778 | >0.999 |
| Vascular endothelial hypertrophy | 3 | 4.0 (3.0–37.0) | 64 | 3.5 (1.0–7.0) | 0.434 | 0.911 |
| Intraluminal macrophage proliferation | 7 | 2.0 (0.0–10.0) | 60 | 4.0 (1.3–7.0) | 0.298 | 0.751 |
| Intraluminal neutrophil proliferation | 10 | 7.5 (0.8–19.8) | 57 | 3.0 (1.0–6.9) | 0.361 | 0.827 |
| Intraluminal eosinophil proliferation | 6 | 5.5 (2.5–6.8) | 61 | 3.0 (1.0–8.5) | 0.655 | >0.999 |
| Intraluminal lymphocyte proliferation | 16 | 2.5 (1.0–8.3) | 51 | 4.0 (2.0–7.0) | 0.282 | 0.751 |
| Intraluminal plasma cell proliferation | 7 | 4.0 (1.0–18.0) | 60 | 3.5 (1.0–7.0) | 0.972 | >0.999 |
| Type II pneumocyte hyperplasia | 61 | 3.0 (1.0–8.0) | 6 | 4.0 (2.3–11.0) | 0.867 | >0.999 |
| Bronchial epithelial cell hyperplasia | 59 | 4.0 (2.0–10.0) | 8 | 2.0 (0.3–4.0) | 0.088 | 0.445 |
| Pleural disease | 3 | 1.0 (0.0–40.0) | 64 | 4.0 (1.3–7.0) | 0.523 | 0.941 |
| Airspace enlargement with fibrosis | 8 | 1.0 (1.0–5.3) | 59 | 4.0 (2.0–9.0) | 0.113 | 0.476 |
| Emphysema | 8 | 1.0 (1.0–3.0) | 59 | 4.0 (2.0–9.0) | 0.035 | 0.295 |
| Bullae | 2 | 41.0 (9.0–73.0) | 65 | 3.0 (1.0–7.0) | 0.062 | 0.390 |

Data are presented as median (interquartile range). False discovery rate (FDR) correction was applied using the Benjamini–Krieger–Yekutieli step-up method, with q < 0.05 considered statistically significant. BALF, bronchoalveolar lavage fluid; HF+, histopathological features present; HF–, histopathological features absent.
