# Supplementary material for: Bronchoalveolar lavage fluid cellular fractions reflect individual histopathological features in interstitial pneumonia
Source: PLoS One. 2026 Oct 30;21(10):e0361768. doi: 10.1371/journal.pone.0361768 (PMC13722173; doi:10.1371/journal.pone.0361768)
Supplement: Supplementary file 3 [file pone.0361768.s007.docx]

**S3 Table. Comparison of BALF eosinophil proportion according to the presence or absence of histopathological features**

| **Histopathological feature** | **n (HF+)** | **BALF Eosinophils, % (HF+)** | **n (HF–)** | **BALF Eosinophils, % (HF–)** | **p value** | **q value** |
| --- | --- | --- | --- | --- | --- | --- |
| Alveolitis | 62 | 3.0 (1.0–6.0) | 5 | 1.0 (1.0–13.0) | 0.694 | 0.838 |
| Fibrosis | 62 | 3.5 (1.0–6.0) | 5 | 1.0 (0.0–4.0) | 0.078 | 0.475 |
| Fibroblastic foci | 40 | 3.0 (1.0–6.0) | 27 | 4.0 (1.0–7.0) | 0.921 | 0.953 |
| UIP pattern | 37 | 3.0 (1.0–6.0) | 30 | 3.5 (1.0–7.3) | 0.947 | 0.953 |
| Bronchiolitis | 17 | 2.0 (1.0–5.0) | 50 | 4.0 (1.0–6.3) | 0.121 | 0.569 |
| Intraluminal organization | 19 | 3.0 (1.0–4.0) | 48 | 3.5 (1.0–6.8) | 0.169 | 0.583 |
| Bronchiectasis | 56 | 3.0 (1.0–6.0) | 11 | 3.0 (1.0–7.0) | 0.850 | 0.953 |
| Honeycombing | 6 | 3.0 (1.0–9.8) | 61 | 3.0 (1.0–6.0) | 0.926 | 0.953 |
| Epithelioid cell granuloma | 6 | 2.5 (0.8–4.5) | 61 | 3.0 (1.0–6.0) | 0.320 | 0.618 |
| Bronchovascular bundle thickening | 52 | 3.5 (1.0–6.0) | 15 | 2.0 (1.0–6.0) | 0.581 | 0.739 |
| Lymphoid hyperplasia | 56 | 3.0 (1.0–6.0) | 11 | 2.0 (1.0–4.0) | 0.292 | 0.618 |
| Lymphoid follicle | 28 | 4.0 (2.0–6.8) | 39 | 3.0 (1.0–6.0) | 0.193 | 0.583 |
| Vascular endothelial hypertrophy | 3 | 21.0 (8.0–63.0) | 64 | 3.0 (1.0–6.0) | 0.002 | 0.038 |
| Intraluminal macrophage proliferation | 7 | 5.0 (1.0–21.0) | 60 | 3.0 (1.0–6.0) | 0.385 | 0.664 |
| Intraluminal neutrophil proliferation | 10 | 5.0 (1.8–6.3) | 57 | 3.0 (1.0–6.0) | 0.475 | 0.717 |
| Intraluminal eosinophil proliferation | 6 | 14.0 (1.8–35.3) | 61 | 3.0 (1.0–6.0) | 0.051 | 0.475 |
| Intraluminal lymphocyte proliferation | 16 | 2.0 (1.0–6.8) | 51 | 4.0 (1.0–6.0) | 0.333 | 0.618 |
| Intraluminal plasma cell proliferation | 7 | 2.0 (1.0–3.0) | 60 | 4.0 (1.0–6.0) | 0.264 | 0.618 |
| Type II pneumocyte hyperplasia | 61 | 3.0 (1.0–6.0) | 6 | 5.0 (1.0–10.3) | 0.526 | 0.739 |
| Bronchial epithelial cell hyperplasia | 59 | 3.0 (1.0–6.0) | 8 | 2.0 (1.0–6.0) | 0.581 | 0.739 |
| Pleural disease | 3 | 1.0 (0.0–6.0) | 64 | 3.0 (1.0–6.0) | 0.306 | 0.618 |
| Airspace enlargement with fibrosis | 8 | 5.5 (1.5–20.8) | 59 | 3.0 (1.0–6.0) | 0.141 | 0.569 |
| Emphysema | 8 | 5.5 (1.0–17.5) | 59 | 3.0 (1.0–6.0) | 0.463 | 0.717 |
| Bullae | 2 | 8.5 (8.0–9.0) | 65 | 3.0 (1.0–6.0) | 0.079 | 0.475 |

Data are presented as median (interquartile range). False discovery rate (FDR) was controlled using the step-up method of Benjamini, Krieger and Yekutieli, with a significance threshold of q < 0.05. BALF, bronchoalveolar lavage fluid; HF+, histopathological features present; HF–, histopathological features absent.
