# Supplementary material for: Bronchoalveolar lavage fluid cellular fractions reflect individual histopathological features in interstitial pneumonia
Source: PLoS One. 2026 Oct 30;21(10):e0361768. doi: 10.1371/journal.pone.0361768 (PMC13722173; doi:10.1371/journal.pone.0361768)
Supplement: Supplementary file 4 [file pone.0361768.s008.docx]

**S4 Table. Sensitivity analysis using Firth penalized maximum likelihood estimation**

| **Variable** | **Primary model (3 variables, maximum likelihood)** | **Firth model (3 variables)** | **Firth model (5 variables)** |
| --- | --- | --- | --- |
| Fibroblastic foci | 0.23 (0.06–0.78); p = 0.018 | 0.26 (0.07–0.82); p = 0.022 | 0.29 (0.08–0.97); p = 0.045 |
| Intraluminal organization | 3.36 (0.80–17.38); p = 0.099 | 2.97 (0.77–13.51); p = 0.114 | 2.64 (0.63–12.64); p = 0.187 |
| Intraluminal lymphocyte proliferation | 5.25 (1.12–38.21); p = 0.035 | 4.26 (1.02–24.77); p = 0.047 | 3.02 (0.66–18.23); p = 0.156 |
| Bronchiolitis | Not entered | Not entered | 3.62 (0.93–17.18); p = 0.064 |
| Epithelioid cell granulomas | Not estimable | Not entered | 1.73 (0.12–261.20); p = 0.729 |

Values are odds ratios with 95% confidence intervals for BALF lymphocytosis, defined as a BALF lymphocyte proportion of at least 15%. Confidence intervals and p values were derived from profile likelihood in the primary model and from penalized profile likelihood in the Firth models.

The primary model was prespecified to include three variables, because the smaller outcome group comprised 29 patients and a limit of 10 events per variable was applied. Epithelioid cell granulomas were present in 6 of 38 patients with BALF lymphocytosis and in none of the 29 patients without it, producing complete separation; when this variable was entered into a conventional maximum likelihood model, the odds ratio diverged to 2.6 × 10¹³ with a standard error of 6.2 × 10⁶. Firth penalized estimation was therefore applied as a sensitivity analysis. In the five-variable Firth model, only fibroblastic foci retained a significant association, whereas the remaining estimates were unstable, supporting retention of the prespecified three-variable model as the primary analysis. Variance inflation factors ranged from 1.11 to 1.20 in the three-variable model and from 1.16 to 1.31 in the five-variable model, indicating that instability reflected the number of events rather than collinearity.

BALF, bronchoalveolar lavage fluid.
