# Supplementary material for: Bronchoalveolar lavage fluid cellular fractions reflect individual histopathological features in interstitial pneumonia
Source: PLoS One. 2026 Oct 30;21(10):e0361768. doi: 10.1371/journal.pone.0361768 (PMC13722173; doi:10.1371/journal.pone.0361768)
Supplement: Supplementary file 5 [file pone.0361768.s009.docx]

**S5 Table. Multivariable analysis using two definitions of BALF lymphocytosis**

| **Variable** | **Cutoff of 15% (primary, 38 vs 29 patients)** | **Cutoff of 18.5% (sensitivity, 36 vs 31 patients)** |
| --- | --- | --- |
| Fibroblastic foci | 0.23 (0.06–0.78); p = 0.018 | 0.17 (0.04–0.58); p = 0.004 |
| Intraluminal organization | 3.36 (0.80–17.38); p = 0.099 | 2.25 (0.54–10.25); p = 0.264 |
| Intraluminal lymphocyte proliferation | 5.25 (1.12–38.21); p = 0.035 | 6.60 (1.40–48.50); p = 0.016 |

Values are odds ratios with 95% confidence intervals derived from profile likelihood. The threshold of 15% was prespecified according to the 2012 American Thoracic Society clinical practice guideline and defines the primary analysis. The threshold of 18.5% was derived post hoc from receiver operating characteristic analysis in the present cohort and is presented as a sensitivity analysis. Estimates for fibroblastic foci and for intraluminal lymphocyte proliferation were directionally consistent across the two thresholds.

BALF, bronchoalveolar lavage fluid.
