# Supplementary material for: Bronchoalveolar lavage fluid cellular fractions reflect individual histopathological features in interstitial pneumonia
Source: PLoS One. 2026 Oct 30;21(10):e0361768. doi: 10.1371/journal.pone.0361768 (PMC13722173; doi:10.1371/journal.pone.0361768)
Supplement: Supplementary file 6 [file pone.0361768.s011.docx]

**Response to Reviewers**

Manuscript PONE-D-26-29295

*Bronchoalveolar lavage fluid cellular fractions reflect individual histopathological features in interstitial pneumonia*

We are grateful for the careful and constructive comments of the reviewers. We are responding to all comments from the three reviewers, together with the requirements raised by the editorial office. We agree with these comments and have addressed each of them in the revised manuscript, tables, and supporting information. All additional analyses were performed on the existing dataset; no patients were added and no histological material was re-examined. The corrections are indicated in the text by visible track changes. Reviewer comments are reproduced below in italics.

We look forward to your review of this revised version.

**Journal requirements**

**Requirement 1. PLOS ONE style and file naming**

The manuscript, title page, and file names have been reformatted according to the PLOS ONE templates.

**Requirement 2. Identifying data in the supporting dataset**

We apologize for submitting a dataset that had not been adequately de-identified. In the revised S1 Dataset, ages are reported in five-year bands, all dates have been removed, and intervals between procedures and durations of follow-up are given as relative values in days. Patient identifiers have been replaced by sequential numbers that bear no relation to the original hospital records, and columns containing personal information have been deleted rather than hidden. The revised file is uploaded with this submission.

**Requirement 3. Citation of previously published work**

No reviewer recommended the citation of specific publications, and no citations have been added on this basis.

**Reviewer 1**

We are grateful to reviewer 1 for a thorough reading and for pressing us on the clinical value of the question we asked. The comments led us to sharpen the framing of the study and to add two analyses that we agree strengthen it.

**Comment 1**

*Although detailed histopathological findings obtained by SLB are important, their primary clinical value lies in disease phenotyping, prediction of treatment response, and prognostic assessment. While the associations between BALF cellular fractions and individual histopathological features are interesting, the clinical significance of correlating BALF findings with each individual pathological feature is limited. It would be more informative to investigate the extent to which BALF cellular profiles, alone or in combination with HRCT findings, contribute to ILD phenotyping, prediction of treatment response, or prognostic stratification.*

We agree that the correspondence between individual features and BALF cellular fractions does not by itself improve clinical outcomes, and we have narrowed the framing of the study accordingly. The manuscript is now built around a single feature of established prognostic importance, fibroblastic foci, and around the question of what BALF adds when histological material is not available.

Following the reviewer’s suggestion, we quantified the contribution of BALF cellular fractions alongside HRCT. In logistic regression models for the presence of fibroblastic foci, the area under the curve was 0.686 for the HRCT pattern alone, 0.764 for the BALF lymphocyte proportion alone, and 0.847 for the two combined. The addition of the BALF lymphocyte proportion to the radiological model was significant on the DeLong test (p = 0.005), and bootstrap resampling with 2000 replications gave a corrected area under the curve of 0.827 for the combined model. This analysis has been added to the Results. We note in the Limitations that these models were developed and evaluated in the same cohort and have not been externally validated.

Prediction of treatment response could not be examined. Treatment in this cohort was not standardized, and serial pulmonary function data sufficient to define a response were not collected. We state this explicitly in the Limitations rather than offer an analysis that the data could not support.

**Comment 2**

*Were there any characteristic BALF findings among patients whose HRCT patterns were discordant with the final multidisciplinary diagnosis? If BALF findings helped predict the final diagnosis in these diagnostically challenging cases, this would provide stronger evidence supporting the clinical utility of BALF as an adjunctive diagnostic tool.*

We examined this question and report the result, which is negative. Taking a probable UIP pattern on HRCT and a final diagnosis of IPF as the concordant pairing, the two were discordant in 7 of the 67 patients. Among the 4 patients with a probable UIP pattern whose final diagnosis was not IPF, BALF lymphocyte proportions were 5.0%, 8.0%, 5.0%, and 78.0%. The three lower values were indistinguishable from the median of 5.5% observed in the 6 patients with a probable UIP pattern and a final diagnosis of IPF. The single patient with marked lymphocytosis received a final diagnosis of CHP and had epithelioid cell granulomas on histology; in that patient BALF was informative, but one patient in four does not support a general claim. Among the 3 patients with IPF whose HRCT pattern was not probable UIP, BALF lymphocyte proportions were 5.0%, 16.0%, and 23.0%, and none suggested IPF.

The same pattern held across the cohort. BALF lymphocyte proportion did not differ significantly among the five final diagnoses (p = 0.306), discrimination between IPF and other diagnoses was limited (area under the curve 0.691, p = 0.068), and it was lower still among the 57 patients whose HRCT pattern was not probable UIP (area under the curve 0.657, p = 0.371).

These results are now reported in a new subsection of the Results, and we draw a conclusion from them in the Discussion. In this cohort, BALF cellular fractions did not help to establish the final diagnosis, yet they were robustly associated with an individual histological feature. We take this contrast to support the premise of the study, namely that the information carried by BALF is expressed more clearly at the level of individual features than at the level of diagnostic labels. We also state that 7 discordant patients are too few to settle the question and that multicenter data would be required.

**Comment 3**

*Histopathological findings were evaluated only as binary variables (present or absent). However, the severity or extent of each pathological feature is likely to have greater biological and clinical relevance. If semi-quantitative grading of histopathological findings is available, analyzing the relationship between the degree of pathological change and BALF cellular fractions would provide more meaningful information.*

We agree that the extent of a feature is likely to carry more biological information than its presence alone. Our decision to use a binary classification was made at the design stage. Grading the extent of a feature depends substantially on subjective judgement, whereas recording a feature as present or absent was expected to yield greater consistency between evaluators, and we gave priority to that consistency across 24 features. We have added this reasoning to the Methods and have listed the resulting constraint in the Limitations.

**Comment 4**

*The study population consisted of several distinct ILD entities, including IPF, NSIP, CTD-ILD, chronic hypersensitivity pneumonitis, and unclassifiable IP... Therefore, the observed associations may simply reflect differences in disease composition rather than direct relationships between BALF cellular fractions and individual histopathological features. Subgroup analyses or analyses adjusted for the final diagnosis would strengthen the validity of the conclusions.*

We have addressed this concern with stratified and adjusted analyses, which are now presented in Table 4. Of the 40 patients with fibroblastic foci, 29 had a histopathological pattern other than UIP or probable UIP, and 31 had a final diagnosis other than IPF, indicating that fibroblastic foci were not restricted to UIP or IPF.

The negative association between fibroblastic foci and BALF lymphocyte proportion persisted in the strata examined. Among the 56 patients whose histopathological pattern was neither UIP nor probable UIP, the median BALF lymphocyte proportion was 14.0% in patients with fibroblastic foci and 29.0% in those without (p = 0.002). The corresponding values were 13.0% versus 29.0% among the 58 patients without a diagnosis of IPF (p = 0.001), 13.0% versus 27.0% among the 44 patients whose pattern was classified as an alternative diagnosis (p = 0.007), and 13.0% versus 32.5% among the 19 patients with idiopathic NSIP (p = 0.006). Among the 23 patients with unclassifiable interstitial pneumonia, the difference was in the same direction but did not reach statistical significance (13.0% versus 47.5%, p = 0.067).

Fibroblastic foci also remained associated with a lower likelihood of BALF lymphocytosis after adjustment for histopathological pattern (odds ratio, 0.27; 95% CI, 0.07–0.95; p = 0.041) and after adjustment for final diagnosis of IPF (odds ratio, 0.22; 95% CI, 0.06–0.79; p = 0.019). Neither histopathological pattern nor final diagnosis of IPF was independently associated with BALF lymphocytosis in these models.

**Comment 5**

*The manuscript reports an ROC-derived cutoff of 18.5% for BALF lymphocyte proportion, whereas the multivariable analysis used the guideline-based cutoff of 15%. The rationale for using two different thresholds should be clarified, and it would be informative to determine whether the results remain consistent when the ROC-derived cutoff is applied.*

Both thresholds are now explained in one place in the Statistical analysis section. The 15% threshold is taken from the 2012 American Thoracic Society clinical practice guideline, was prespecified, and defines the primary analysis. The 18.5% threshold was derived post hoc from ROC analysis in this cohort and is used only to describe the discrimination of fibroblastic foci. We also state the resulting group sizes, 38 and 29 patients at 15% and 36 and 31 patients at 18.5%, since the change in group composition is otherwise difficult to follow.

We repeated the multivariable analysis using the 18.5% threshold as the outcome and report it as S5 Table. Fibroblastic foci were associated with a lower likelihood of lymphocytosis (odds ratio 0.17, 95% CI 0.04 to 0.58, p = 0.004) and intraluminal lymphocyte proliferation with a higher likelihood (odds ratio 6.60, 95% CI 1.40 to 48.50, p = 0.016), consistent in direction with the primary analysis.

**Reviewer 2**

We thank the reviewer for a précis of the methodological issues that was both accurate and economical. Each point is addressed below.

**Comment 1. Potential confounding by ILD subtype**

*The cohort includes multiple ILD subtypes (IPF, NSIP, CTD-ILD, CHP, and unclassifiable IP), each with distinct BAL cellular profiles and histopathology. Please clarify whether the association between BAL lymphocyte proportion and fibroblastic foci is independent of diagnosis by adjusting for disease subtype or performing subgroup/sensitivity analyses.*

Please see our response to Reviewer 1, Comment 4. In brief, the association persisted after exclusion of patients with a UIP or probable UIP pattern and of patients with IPF, in each subgroup examined, and after adjustment for histopathological pattern and for a final diagnosis of IPF. The results are presented in Table 4.

**Comment 2. Multivariable analysis**

*Please provide additional details on the logistic regression model, including variable selection, assessment of multicollinearity, and model stability given the relatively small sample size.*

Our description of the model was insufficient, and we have expanded it. On variable selection, the number of variables entered was fixed in advance at three, since the smaller outcome group comprised 29 patients and a limit of 10 events per variable was applied. The three variables with the smallest univariate p values were selected: fibroblastic foci (p = 0.001), intraluminal organization (p = 0.006), and intraluminal lymphocyte proliferation (p = 0.008). Bronchiolitis (p = 0.022) and epithelioid cell granulomas (p = 0.032) were excluded by this rule, and Table 3 now carries a footnote stating that blank cells denote variables that were not entered.

On inference, the odds ratios, confidence intervals, and p values reported in the originally submitted manuscript were derived from profile likelihood rather than from Wald statistics, but this was not stated. We have now specified it in the Methods.

On multicollinearity, pairwise phi coefficients among the three variables ranged from −0.361 to +0.269, variance inflation factors from 1.11 to 1.20, and the condition number of the design matrix was 4.0, giving no indication that collinearity affected the estimates.

On stability, we added a sensitivity analysis using Firth penalized estimation, reported in S4 Table and described in our response to Reviewer 3, Major point 2.

**Comment 3. Histopathological scoring**

*Histopathological features were analyzed as binary variables (present/absent). Please justify this approach, particularly for fibroblastic foci, where semiquantitative scoring may provide more informative associations.*

Our reasoning is given in the response to Reviewer 1, Comment 3, and has been added to the Methods and the Limitations. In short, recording each feature as present or absent was expected to yield greater consistency between evaluators than graded scoring, and a binary classification was adopted at the design stage for that reason.

**Comment 4. BAL to SLB interval**

*The median interval between BAL and surgical lung biopsy was 43 days (IQR 18–75). Please discuss the potential impact of this interval on the observed associations.*

We understand this concern to be that the two specimens may not represent the same disease state when the interval between them is long. Two aspects of the study design limit this possibility. No patient was receiving treatment for interstitial pneumonia at the time of BAL, so treatment-related change during the interval is not a consideration, and 6 patients in whom disease progression was documented between the two procedures had already been excluded during case selection. In addition, the features examined here, in particular fibroblastic foci, represent structural remodelling that develops over months to years rather than weeks.

We nonetheless acknowledge that change in disease state during the interval cannot be excluded, and we have added this to the Limitations. We also note that surgical lung biopsy preceded bronchoalveolar lavage in 2 patients; the interval is reported as an absolute value, and this is now stated in the footnote to Table 1.

**Comment 5. Interpretation of findings**

*The conclusions occasionally imply predictive capability. Given the retrospective design and lack of external validation, I recommend using more cautious language (e.g. "associated with" or "may provide complementary information") rather than suggesting BAL can reliably infer histopathological findings.*

We accept this without reservation. The words surrogate and predict have been removed throughout, including from the Introduction, where the aim of the study was previously framed in terms of a surrogate for histopathological features. The abstract, Results, Discussion, and Conclusions now state that BALF cellular fractions are associated with histopathological features and may provide complementary information. The Limitations state that the models were developed and evaluated in the same cohort without external validation.

**Reviewer 3**

We are grateful to reviewer 3 for a review that was unusually specific about what would settle each question. The reviewer was correct that all of the concerns could be addressed with the existing dataset, and we have done so.

**Major point 1. Whether the finding restates a pattern-level observation**

*Fibroblastic foci are what define UIP, and low BAL lymphocytes in UIP and IPF has been known for a long time... Please repeat the analysis adjusting for pathological pattern, or stratifying by it, and ideally by IPF versus non IPF as well. If the association holds up, the paper is much stronger. If it fades, please say so and rewrite the interpretation accordingly.*

This was the most important question raised in the review, and we tested it as the reviewer proposed. The association holds.

The cohort was suitable for these analyses because fibroblastic foci were not confined to UIP. Among the 40 patients with fibroblastic foci, 29 had a histopathological pattern other than UIP or probable UIP, and 31 had a final diagnosis other than IPF. In the stratified analyses below, the first value represents the median BALF lymphocyte proportion in patients with fibroblastic foci and the second represents that in patients without fibroblastic foci. When UIP and probable UIP patterns were excluded, the corresponding values were 14.0% and 29.0% (n = 56, p = 0.002). When patients with IPF were excluded, the values were 13.0% and 29.0% (n = 58, p = 0.001). Among patients whose histopathological pattern was classified as an alternative diagnosis, the values were 13.0% and 27.0% (n = 44, p = 0.007). In the idiopathic NSIP subgroup, the values were 13.0% and 32.5% (n = 19, p = 0.006). Among patients with unclassifiable IP, the difference was in the same direction but did not reach statistical significance (13.0% vs. 47.5%; n = 23, p = 0.067).

In the adjusted models, fibroblastic foci remained independently associated with a lower likelihood of BALF lymphocytosis after adjustment for histopathological pattern (odds ratio, 0.27; 95% CI, 0.07–0.95; p = 0.041) and after adjustment for final diagnosis of IPF (odds ratio, 0.22; 95% CI, 0.06–0.79; p = 0.019). In contrast, neither histopathological pattern (odds ratio, 0.55; p = 0.462) nor final diagnosis (odds ratio, 1.24; p = 0.798) was significantly associated with the outcome. As noted in Table 4 and the Discussion, these adjusted analyses should be interpreted cautiously because they may involve overadjustment: fibroblastic foci contribute to the definition of the UIP pattern. Therefore, the stratified comparisons provide more informative evidence for the robustness of the association. A new paragraph has been added to the Discussion to explain this rationale.

**Major point 2. The logistic regression and complete separation**

*Five variables were significant on univariate testing, but only three show up in the multivariable columns of Table 3. What happened to bronchiolitis and epithelioid cell granulomas? I suspect the granuloma variable caused complete separation... If that is what happened, please say so, and consider Firth regression, which handles this. I would also ask the authors to soften the wording around intraluminal lymphocyte proliferation.*

The reviewer’s inference is correct, and we should have stated it.

Two reasons account for the three variables in the multivariable columns, and both are now given in the Methods. First, the number of variables was fixed in advance at three on the basis of 10 events per variable, and the three with the smallest univariate p values were selected, which excluded bronchiolitis and epithelioid cell granulomas. Second, epithelioid cell granulomas could not have been estimated in any case: they were present in 6 of the 38 patients with BALF lymphocytosis and in none of the 29 patients without it, and entering this variable into a conventional maximum likelihood model causes the odds ratio to diverge to 2.6 × 10¹³ with a standard error of 6.2 × 10⁶. Fibroblastic foci and epithelioid cell granulomas never co-occurred in this cohort (phi = −0.382, p = 0.003), which contributes to the separation.

We added the Firth analysis the reviewer suggested, now S4 Table. With all five variables entered simultaneously, fibroblastic foci retained a significant association (odds ratio 0.29, 95% CI 0.08 to 0.97, p = 0.045), while bronchiolitis (3.62, 0.93 to 17.18, p = 0.064), intraluminal organization (2.64, 0.63 to 12.64, p = 0.187), intraluminal lymphocyte proliferation (3.02, 0.66 to 18.23, p = 0.156), and epithelioid cell granulomas (1.73, 0.12 to 261.20, p = 0.729) did not. Variance inflation factors in this model ranged from 1.16 to 1.31, so the instability reflects the number of events rather than collinearity. We therefore retain the prespecified three-variable model as the primary analysis and present the five-variable Firth model as a sensitivity analysis that demonstrates what happens when the model is expanded beyond what the data support.

We have also softened the wording on intraluminal lymphocyte proliferation, as the reviewer asked. Applying Firth estimation to the three-variable model reduces the odds ratio to 4.26 (1.02 to 24.77), and in the five-variable model the association is lost. The Results now report the numbers on which the estimate rests, 14 patients against 2, without describing it as an independent association, and the Discussion states that it should be regarded as exploratory.

**Major point 3. The clinical claim**

*The authors suggest BAL could stand in for biopsy in inferring pathology. But an AUC of 0.76 with 65% sensitivity means about a third of patients with fibroblastic foci would be missed... More to the point, survival was not significantly different when patients were split at the 18.5% BAL cutoff (p = 0.104), even though it was clearly different when split by the histology itself (p = 0.003). That is an important result and it currently passes almost without comment.*

We agree on both counts and have rewritten the claims accordingly. The abstract and Conclusions no longer suggest that BALF can stand in for biopsy; they state that BALF cellular fractions are associated with histopathological features and may provide complementary information, and the Conclusions note that the moderate discriminatory performance observed here indicates that BALF is unlikely to substitute for histological assessment.

The discrepancy between the two survival analyses now has a paragraph of its own in the Discussion. Stratification by the histological feature separated the survival curves clearly, whereas stratification by BALF lymphocyte proportion did not, at either 18.5% (p = 0.104) or the guideline threshold of 15% (p = 0.413), and we report the latter figure as well. We interpret this, as the reviewer implies, as direct evidence that BALF captures the presence of fibroblastic foci only partially.

**Major point 4. Findings resting on very few patients**

*The eosinophil and vascular endothelial hypertrophy result (q = 0.038) comes from 3 patients with the feature. FDR correction helps with the multiple testing problem, but it cannot make an estimate from three patients reliable... I would go further with the eosinophil finding and give it less prominence.*

The reviewer is right that false discovery rate correction addresses multiplicity and not the instability of an estimate based on three patients. We have reduced the prominence of the eosinophil finding: the sentence reporting it in the Results now states that the feature was present in only 3 patients, and the discussion of it has been removed. The Limitations identify vascular endothelial hypertrophy, pleural disease, bullae, and epithelioid cell granulomas as features observed in too few patients to support reliable estimation, and note that they should be regarded as hypothesis generating.

**Major point 5. Details of the survival analysis**

*Saying survival ran from SLB to censoring does not tell me what counted as an event. Was it death from any cause, or respiratory death? How many deaths were there in each group? What was the median follow up? Were any patients transplanted, and if so how were they handled?*

The original sentence was not a usable definition, and we have replaced it. The event was death from any cause, and survival time was measured from the date of surgical lung biopsy to death or to the date of last confirmed follow-up. Nineteen of the 67 patients died, comprising 16 of the 40 with fibroblastic foci (40.0%) and 3 of the 27 without (11.1%). Median survival was 8.3 years in the presence of fibroblastic foci and was not reached in their absence. The median duration of follow-up by the reverse Kaplan–Meier method was 7.5 years, with observed follow-up ranging from 0.2 to 15.9 years. No patient underwent lung transplantation during follow-up.

**Minor points**

Two cutoffs. Both are now explained together in the Statistical analysis section, including the reason for each and the resulting group sizes of 38 and 29 at 15% and 36 and 31 at 18.5%.

Overlapping roles of one evaluator. We have made the arrangement explicit. HRCT images and histopathological specimens were reviewed in separate sessions using de-identified material, so that a given image could not be linked to the corresponding specimen or patient; a judgement made in one review could not therefore inform the other in an individual patient. That said, S.H. participated in the radiological review, the histopathological review, and the multidisciplinary discussion, and the multidisciplinary discussion integrates all available information by design. The Limitations now state that complete independence of assessment cannot be guaranteed.

Title of Table 3. Changed to “Univariate and multivariable analysis of factors associated with BALF lymphocytosis (≥ 15%)”.

Line 55. “Extenuating” has been replaced by “progressive”, which was the intended word.

Lines 317 to 319. Rewritten as “whether reduced lymphocyte numbers contribute to the formation of fibroblastic foci or the reverse”.

Line 244. The percent sign has been added.

Line 251. We will confirm the hyphenation of “non-invasive” at proof stage.

Line 144. The initials are now rendered as S.H. and Y.N., matching usage elsewhere.

**Closing**

One correction should be noted that no reviewer raised. During preparation of the revised dataset we found that one value had not been carried across correctly when Table 1 was compiled, and the median DLCO is 73.1% of predicted rather than 72.3%. DLCO was not used in any analysis, so no result is affected. The value has been corrected in Table 1.

We thank the reviewers again for the care they gave the manuscript.
