# Supplementary material for: Bronchoalveolar lavage fluid cellular fractions reflect individual histopathological features in interstitial pneumonia
Source: PLoS One. 2026 Oct 30;21(10):e0361768. doi: 10.1371/journal.pone.0361768 (PMC13722173; doi:10.1371/journal.pone.0361768)
Supplement: Peer review history

# Peer review: Decision Letter 0

**DOI:** 10.1371/journal.pone.0361768.r001

**Authors:** Rohit Gaurav

20 Jul 2026

Dear Dr. Miyoshi,

Thank you for submitting your manuscript to PLOS ONE. After careful consideration, we feel that it has merit but does not fully meet PLOS ONE’s publication criteria as it currently stands. Therefore, we invite you to submit a revised version of the manuscript that addresses the points raised during the review process.

Please submit your revised manuscript bySep 18 2026 11:59PM. If you will need more time than this to complete your revisions, please reply to this message or contact the journal office at plosone@plos.org. When you're ready to submit your revision, log on to https://www.editorialmanager.com/pone/ and select the 'Submissions Needing Revision' folder to locate your manuscript file.

- A letter that responds to each point raised by the academic editor and reviewer(s). You should upload this letter as a separate file labeled 'Response to Reviewers'.
- A marked-up copy of your manuscript that highlights changes made to the original version. You should upload this as a separate file labeled 'Revised Manuscript with Track Changes'.
- An unmarked version of your revised paper without tracked changes. You should upload this as a separate file labeled 'Manuscript'.

If you would like to make changes to your financial disclosure, please include your updated statement in your cover letter. Guidelines for resubmitting your figure files are available below the reviewer comments at the end of this letter.

If applicable, we recommend that you deposit your laboratory protocols in protocols.io to enhance the reproducibility of your results. Protocols.io assigns your protocol its own identifier (DOI) so that it can be cited independently in the future. For instructions see: https://journals.plos.org/plosone/s/submission-guidelines#loc-laboratory-protocols. Additionally, PLOS ONE offers an option for publishing peer-reviewed Lab Protocol articles, which describe protocols hosted on protocols.io. Read more information on sharing protocols at https://plos.org/protocols?utm_medium=editorial-email&utm_source=authorletters&utm_campaign=protocols.

As the corresponding author, your ORCID iD is verified in the submission system and will appear in the published article. PLOS supports the use of ORCID, and we encourage all coauthors to register for an ORCID iD and use it as well. Please encourage your coauthors to verify their ORCID iD within the submission system before final acceptance, as unverified ORCID iDs will not appear in the published article. *Only*  the individual author can complete the verification step; PLOS staff *cannot*  verify ORCID iDs on behalf of authors.

We look forward to receiving your revised manuscript.

Kind regards,

Rohit Gaurav, PhD

Academic Editor

PLOS One

Journal Requirements:

When submitting your revision, we need you to address these additional requirements.

1. Please ensure that your manuscript meets PLOS ONE's style requirements, including those for file naming. The PLOS ONE style templates can be found at

https://journals.plos.org/plosone/s/file?id=wjVg/PLOSOne_formatting_sample_main_body.pdf and

https://journals.plos.org/plosone/s/file?id=ba62/PLOSOne_formatting_sample_title_authors_affiliations.pdf

2. We note that there is identifying data in the Supporting Information file S1_Dataset.xlsx. Due to the inclusion of these potentially identifying data, we have removed this file from your file inventory. Prior to sharing human research participant data, authors should consult with an ethics committee to ensure data are shared in accordance with participant consent and all applicable local laws.

Data sharing should never compromise participant privacy. It is therefore not appropriate to publicly share personally identifiable data on human research participants. The following are examples of data that should not be shared:

-Name, initials, physical address

-Ages more specific than whole numbers

-Internet protocol (IP) address

-Specific dates (birth dates, death dates, examination dates, etc.)

-Contact information such as phone number or email address

-Location data

-ID numbers that seem specific (long numbers, include initials, titled “Hospital ID”) rather than random (small numbers in numerical order)

Data that are not directly identifying may also be inappropriate to share, as in combination they can become identifying. For example, data collected from a small group of participants, vulnerable populations, or private groups should not be shared if they involve indirect identifiers (such as sex, ethnicity, location, etc.) that may risk the identification of study participants.

Additional guidance on preparing raw data for publication can be found in our Data Policy (https://journals.plos.org/plosone/s/data-availability#loc-human-research-participant-data-and-other-sensitive-data) and in the following article: http://www.bmj.com/content/340/bmj.c181.long.

Please remove or anonymize all personal information age, ensure that the data shared are in accordance with participant consent, and re-upload a fully anonymized data set. Please note that spreadsheet columns with personal information must be removed and not hidden as all hidden columns will appear in the published file.

3. If the reviewer comments include a recommendation to cite specific previously published works, please review and evaluate these publications to determine whether they are relevant and should be cited. There is no requirement to cite these works unless the editor has indicated otherwise.

[Note: HTML markup is below. Please do not edit.]

Reviewers' comments:

Reviewer's Responses to Questions

**Comments to the Author**

1. Is the manuscript technically sound, and do the data support the conclusions?

Reviewer #1: Partly

Reviewer #2: Partly

Reviewer #3: Partly

**********

2. Has the statistical analysis been performed appropriately and rigorously?

Reviewer #1: Yes

Reviewer #2: Yes

Reviewer #3: No

**********

3. Have the authors made all data underlying the findings in their manuscript fully available?

The PLOS Data policy

Reviewer #1: No

Reviewer #2: Yes

Reviewer #3: Yes

**********

4. Is the manuscript presented in an intelligible fashion and written in standard English?

Reviewer #1: Yes

Reviewer #2: Yes

Reviewer #3: No

**********

Reviewer #1: The authors investigated the associations between bronchoalveolar lavage fluid (BALF) cellular fractions and histopathological findings obtained by surgical lung biopsy (SLB) in patients with interstitial lung disease (ILD). This study provides an interesting and unique perspective; however, the sample size is relatively small, and the clinical significance of the primary research question remains unclear. Although the observed associations between BALF cellular profiles and individual histopathological features are of scientific interest, the manuscript does not sufficiently demonstrate how these findings improve clinically relevant outcomes, such as disease classification, prediction of treatment response, or prognosis. Furthermore, the heterogeneous study population raises concerns that the observed associations may be confounded by differences among ILD subtypes. Addressing the following issues would substantially strengthen the manuscript.

Comments

1. Although detailed histopathological findings obtained by SLB are important, their primary clinical value lies in disease phenotyping, prediction of treatment response (e.g., corticosteroids or antifibrotic agents), and prognostic assessment. While the associations between BALF cellular fractions and individual histopathological features are interesting, the clinical significance of correlating BALF findings with each individual pathological feature is limited. It would be more informative to investigate the extent to which BALF cellular profiles, alone or in combination with HRCT findings, contribute to ILD phenotyping, prediction of treatment response, or prognostic stratification.

2. Were there any characteristic BALF findings among patients whose HRCT patterns were discordant with the final multidisciplinary diagnosis? If BALF findings helped predict the final diagnosis in these diagnostically challenging cases, this would provide stronger evidence supporting the clinical utility of BALF as an adjunctive diagnostic tool.

3. Histopathological findings were evaluated only as binary variables (present or absent). However, the severity or extent of each pathological feature is likely to have greater biological and clinical relevance. If semi-quantitative grading of histopathological findings is available, analyzing the relationship between the degree of pathological change and BALF cellular fractions would provide more meaningful information.

4. The study population consisted of several distinct ILD entities, including IPF, NSIP, CTD-ILD, chronic hypersensitivity pneumonitis, and unclassifiable IP, each of which is known to have different BALF cellular profiles and histopathological characteristics. Therefore, the observed associations may simply reflect differences in disease composition rather than direct relationships between BALF cellular fractions and individual histopathological features. Subgroup analyses or analyses adjusted for the final diagnosis would strengthen the validity of the conclusions.

5. The manuscript reports an ROC-derived cutoff of 18.5% for BALF lymphocyte proportion, whereas the multivariable analysis used the guideline-based cutoff of 15%. The rationale for using two different thresholds should be clarified, and it would be informative to determine whether the results remain consistent when the ROC-derived cutoff is applied.

Reviewer #2: This manuscript addresses an important clinical question regarding the relationship between BALF cellular fractions and histopathological features in interstitial pneumonia. The study is well organized, the analyses are generally appropriate for an exploratory retrospective study, and the findings are clinically relevant. However, several methodological and statistical issues should be addressed before the conclusions can be fully supported.

1. Potential confounding by ILD subtype: The cohort includes multiple ILD subtypes (IPF, NSIP, CTD-ILD, CHP, and unclassifiable IP), each with distinct BAL cellular profiles and histopathology. Please clarify whether the association between BAL lymphocyte proportion and fibroblastic foci is independent of diagnosis by adjusting for disease subtype or performing subgroup/sensitivity analyses.

2. Multivariable analysis: Please provide additional details on the logistic regression model, including variable selection, assessment of multicollinearity, and model stability given the relatively small sample size.

3. Histopathological scoring: Histopathological features were analyzed as binary variables (present/absent). Please justify this approach, particularly for fibroblastic foci, where semiquantitative scoring may provide more informative associations.

4. BAL–SLB interval: The median interval between BAL and surgical lung biopsy was 43 days (IQR 18–75). Please discuss the potential impact of this interval on the observed associations.

5. Interpretation of findings: The conclusions occasionally imply predictive capability. Given the retrospective design and lack of external validation, I recommend using more cautious language (e.g., "associated with" or "may provide complementary information") rather than suggesting BAL can reliably infer histopathological findings.

Reviewer #3: The authors looked at 67 patients with interstitial pneumonia who had both a BAL and a surgical lung biopsy, and asked whether the cell counts in the BAL fluid line up with 24 individual features seen on histology. They found that lymphocyte proportion was lower when fibroblastic foci were present, and higher with bronchiolitis, intraluminal organization, and granulomas. Patients with fibroblastic foci did worse.

I think this is a useful question to ask. Having BAL and SLB in the same patients is not easy to come by, and scoring individual features instead of just lumping cases into patterns is a good idea. I was also glad to see the FDR correction, which many papers in this space skip.

That said, a few of the conclusions go further than the data currently allow. All of my concerns can be dealt with using the dataset the authors already have. I recommend major revision.

Major points

My main concern is that the headline finding may not be telling us anything new. Fibroblastic foci are what define UIP, and low BAL lymphocytes in UIP and IPF has been known for a long time. The authors show this themselves in Fig 2a, where lymphocyte proportion is clearly lower in the UIP and probable UIP cases. So when they report that lymphocytes are lower when fibroblastic foci are present, I cannot tell whether this is a genuine link to the individual feature or just the old pattern level finding in a different guise. Since the whole point of the paper is that BAL reflects individual features, this needs sorting out. Please repeat the analysis adjusting for pathological pattern, or stratifying by it, and ideally by IPF versus non IPF as well. If the association holds up, the paper is much stronger. If it fades, please say so and rewrite the interpretation accordingly.

The logistic regression needs more explanation. Five variables were significant on univariate testing, but only three show up in the multivariable columns of Table 3. What happened to bronchiolitis and epithelioid cell granulomas? I suspect the granuloma variable caused complete separation, since it was 6/38 versus 0/29 and there are no patients in one cell. If that is what happened, please say so, and consider Firth regression, which handles this. I would also ask the authors to soften the wording around intraluminal lymphocyte proliferation. An odds ratio of 5.25 with a confidence interval running from 1.12 to 38.21, based on 14 patients versus 2, is not a stable estimate, and calling it independently associated gives the reader more confidence than the numbers deserve.

The clinical pitch is stronger than the results support. The authors suggest BAL could stand in for biopsy in inferring pathology. But an AUC of 0.76 with 65% sensitivity means about a third of patients with fibroblastic foci would be missed, and that is the one feature they argue matters most for prognosis. More to the point, survival was not significantly different when patients were split at the 18.5% BAL cutoff (p = 0.104), even though it was clearly different when split by the histology itself (p = 0.003). That is an important result and it currently passes almost without comment. Please address it directly, and pull back the abstract and conclusions to say BAL adds useful supporting information rather than that it can substitute for knowing the histology.

Some findings rest on very few patients. The eosinophil and vascular endothelial hypertrophy result (q = 0.038) comes from 3 patients with the feature. FDR correction helps with the multiple testing problem, but it cannot make an estimate from three patients reliable. The same goes for bullae (n = 2), pleural disease (n = 3), and granulomas (n = 6). Please flag these as hypothesis generating. I would go further with the eosinophil finding and give it less prominence, so it is not read as sitting alongside the lymphocyte results in reliability.

The survival analysis is missing some basic details. Saying survival ran from SLB to censoring does not tell me what counted as an event. Was it death from any cause, or respiratory death? How many deaths were there in each group? What was the median follow up? Were any patients transplanted, and if so how were they handled?

Minor points

Two different cutoffs are used for lymphocytosis, 15% from the ATS guideline and 18.5% from the ROC curve, and the group sizes change from 38/29 to 36/31 as a result. This is reasonable but hard to follow. Please explain both in one place in the Methods.

S.H. read the HRCT scans and was also one of the two pathologists who reviewed the histology and settled the final diagnosis. Please make this clear and mention it as a limitation.

Table 3 is titled as a multivariate analysis, but most of it is univariate comparisons across 27 variables. The title should reflect both.

Line 55: "extenuating" does not seem to be the intended word. Perhaps "progressive"?

Lines 317 to 319: "whether it contributes to fibroblastic or vice versa" is missing a word, presumably "fibroblastic foci formation".

Line 244: a percent sign is missing after 3.0.

Line 251: "non invasive" is split across lines without a hyphen in the PDF. Worth checking at proofs.

Line 144: S.H and Y.N appear without periods, unlike elsewhere in the text.

**********

what does this mean?). If published, this will include your full peer review and any attached files.

If you choose “no”, your identity will remain anonymous but your review may still be made public.

**Do you want your identity to be public for this peer review?**  For information about this choice, including consent withdrawal, please see our Privacy Policy

Reviewer #1: **Yes:**  Masato Karayama

Reviewer #2: No

Reviewer #3: **Yes:**  Nihal S Mulla

**********

[NOTE: If reviewer comments were submitted as an attachment file, they will be attached to this email and accessible via the submission site. Please log into your account, locate the manuscript record, and check for the action link "View Attachments". If this link does not appear, there are no attachment files.]

To ensure your figures meet our technical requirements, please review our figure guidelines: https://journals.plos.org/plosone/s/figures

You may also use PLOS’s free figure tool, NAAS, to help you prepare publication quality figures: https://journals.plos.org/plosone/s/figures#loc-tools-for-figure-preparation.

NAAS will assess whether your figures meet our technical requirements by comparing each figure against our figure specifications.

# Author comment: Author response to Decision Letter 1

**DOI:** 10.1371/journal.pone.0361768.r002

14 Sep 2026

Our point-by-point response to the editor and reviewers is uploaded as a separate file labeled "Response to Reviewers".

Attachment Submitted filename: Response_to_Reviewers.docx

# Editor report: Decision Letter 1

**DOI:** 10.1371/journal.pone.0361768.r003

**Authors:** Rohit Gaurav

16 Oct 2026

Bronchoalveolar lavage fluid cellular fractions reflect individual histopathological features in interstitial pneumonia

PONE-D-26-29295R1

Dear Dr. Miyoshi,

We’re pleased to inform you that your manuscript has been judged scientifically suitable for publication and will be formally accepted for publication once it meets all outstanding technical requirements.

Within one week, you’ll receive an e-mail detailing the required amendments. When these have been addressed, you’ll receive a formal acceptance letter and your manuscript will be scheduled for publication.

An invoice will be generated when your article is formally accepted. Please note, if your institution has a publishing partnership with PLOS and your article meets the relevant criteria, all or part of your publication costs will be covered. Please make sure your user information is up-to-date by logging into Editorial Manager at Editorial Manager® and clicking the ‘Update My Information' link at the top of the page. For questions related to billing, please contact billing support.

If your institution or institutions have a press office, please notify them about your upcoming paper to help maximize its impact. If they’ll be preparing press materials, please inform our press team as soon as possible -- no later than 48 hours after receiving the formal acceptance. Your manuscript will remain under strict press embargo until 2 pm Eastern Time on the date of publication. For more information, please contact onepress@plos.org.

Kind regards,

Rohit Gaurav, PhD

Academic Editor

PLOS One

Additional Editor Comments (optional):

Reviewers' comments:

# Editor report: Acceptance letter

**DOI:** 10.1371/journal.pone.0361768.r004

**Authors:** Rohit Gaurav

PONE-D-26-29295R1

PLOS One

Dear Dr. Miyoshi,

I'm pleased to inform you that your manuscript has been deemed suitable for publication in PLOS One. Congratulations! Your manuscript is now being handed over to our production team.

At this stage, our production department will prepare your paper for publication. This includes ensuring the following:

* All references, tables, and figures are properly cited

* All relevant supporting information is included in the manuscript submission,

* There are no issues that prevent the paper from being properly typeset

You will receive further instructions from the production team, including instructions on how to review your proof when it is ready. Please keep in mind that we are working through a large volume of accepted articles, so please give us a few days to review your paper and let you know the next and final steps.

Lastly, if your institution or institutions have a press office, please let them know about your upcoming paper now to help maximize its impact. If they'll be preparing press materials, please inform our press team within the next 48 hours. Your manuscript will remain under strict press embargo until 2 pm Eastern Time on the date of publication. For more information, please contact onepress@plos.org.

You will receive an invoice from PLOS for your publication fee after your manuscript has reached the completed accept phase. If you receive an email requesting payment before acceptance or for any other service, this may be a phishing scheme. Learn how to identify phishing emails and protect your accounts at https://explore.plos.org/phishing.

If we can help with anything else, please email us at customercare@plos.org.

Thank you for submitting your work to PLOS One and supporting open access.

Kind regards,

PLOS One Editorial Office Staff

on behalf of

Dr. Rohit Gaurav

Academic Editor

PLOS One
